# Supplementary material for: The HIF target MAFF promotes tumor invasion and metastasis through IL11 and STAT3 signaling
Source: Nat Commun. 2021 Jul 14;12:4308. doi: 10.1038/s41467-021-24631-6 (PMC8280233; doi:10.1038/s41467-021-24631-6)
Supplement: Supplementary file 1 — Supplementary Information [file 41467_2021_24631_MOESM1_ESM.pdf]

Supplementary Information

The HIF target MAFF promotes tumor invasion and metastasis through IL11 and STAT3 signaling

Eui Jung Moon, Stephano S Mello, Caiyun G Li, Jen-Tsan Chi, Kaushik Thakkar, Jacob G. Kirkland, Edward L. Lagory, Ik Jae Lee, Anh N Diep, Yu Miao, Marjan Rafat, Marta Vilalta, Laura Castellini, Adam J Krieg, Edward E Graves, Laura D Attardi, Amato J Giaccia

Corresponding author: Amato J Giaccia, giaccia@stanford.edu

Supplementary Figure 1.

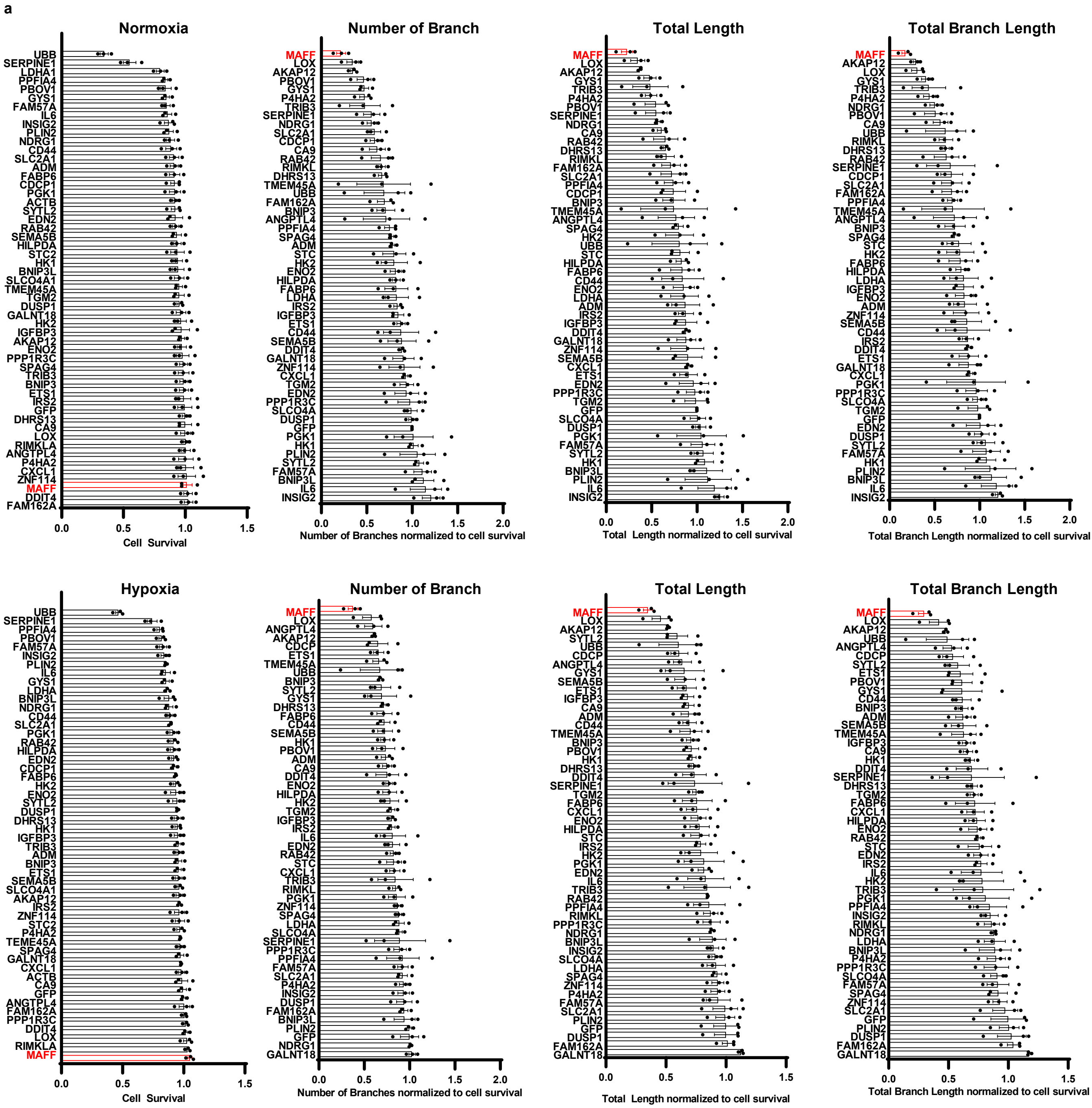

Supplementary Figure 1. Hypoxia-induced MAFF regulates tumor invasion and acts as a prognostic indicator

Using esiRNA library, 50 hypoxia-induced genes were evaluated for their effect on cell survival and invasion under normoxia (a) and hypoxia (b). Cell survival was measured using SRB assay (*Promega*) while invasion assay was performed on collagen I coated 96 well plate. Cell invasion was determined by measuring "number of branch", "total length", and "total branch length" using ImageJ. Graphs represent the mean per group and error bars represent the SEM. *n*=3 biological replicates. One-Way ANOVA with multiple comparisons was used to determine the statistical significance of MAFF knockdown compared to GFP control (Normoxia: Cell survival: ns, Number of Branch: p=0.0003, Total Length: p=0.0066, Total Branch Length: p=0.0035. Hypoxia: Cell Survival: ns, Number of Branch: p=0.0003, Total Length: p=0.0001, Total Branch Length: p=0.0005). ns: not significant.

Supplementary Figure 2.

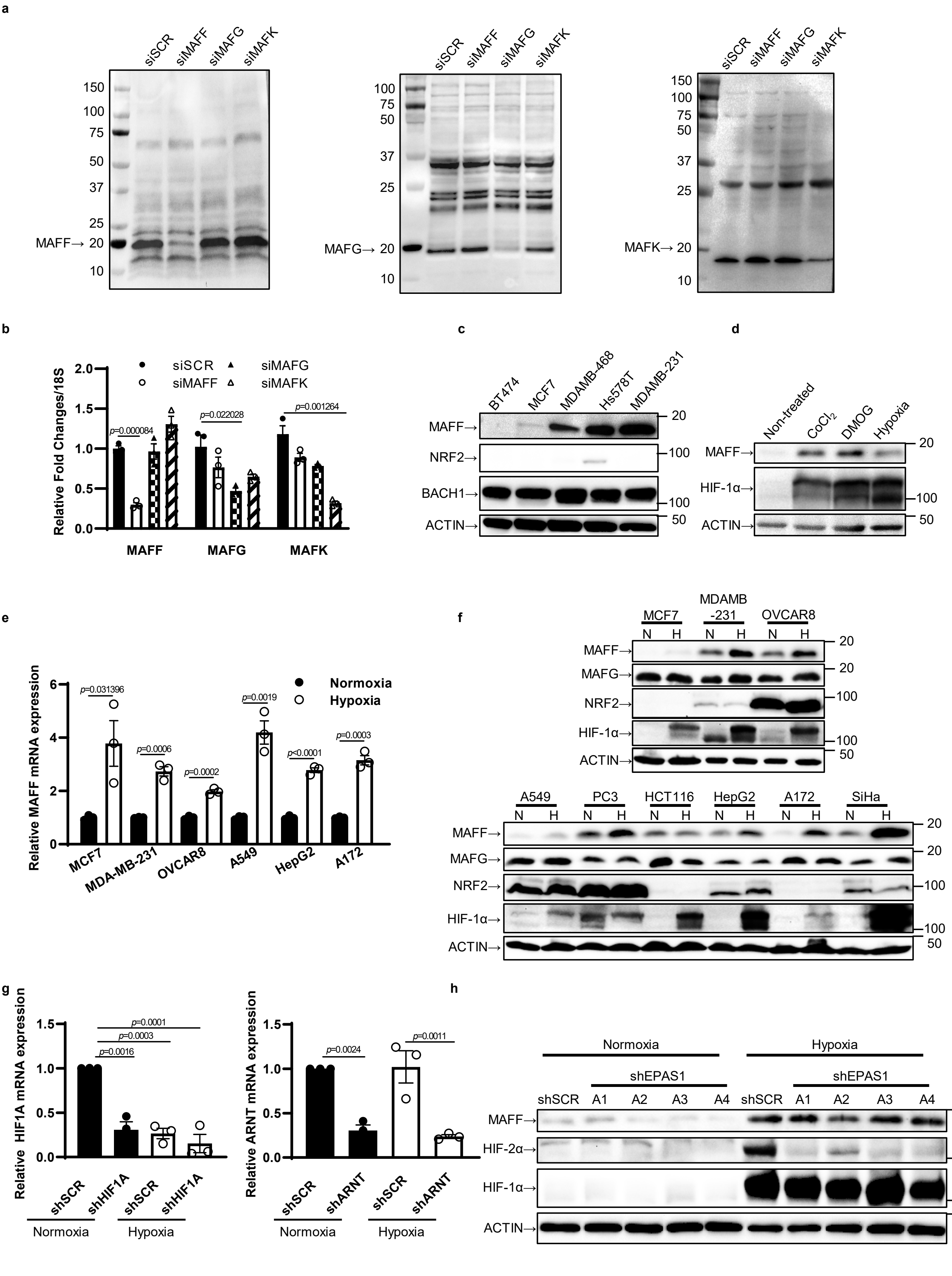

Supplementary Figure 2. MAFF induction under hypoxia is regulated by HIF-1

(a) MAFF antibody specificity was confirmed by knocking down *MAFF* as well as other small MAF family, *MAFG* and *MAFK* using siRNA (*Horizon Discovery*). MAFG and MAFK antibodies also showed specificity to each protein. (b) mRNA expression of *MAFF*, *MAFG*, and *MAFK* were determined after siRNA knockdown. *n*=3 biological replicates. Multiple Unpaired *t*-test. (c) Basal expression of MAFF protein was high in more aggressive and metastatic breast cancer cell lines compared to non-metastatic cells. (d) Treatment with chemical HIF inducers, $CoCl_2$ and DMOG, increased MAFF expression in MDA-MB-231 cells. (e-f) In breast (MCF7, MDA-MB-231), ovarian (OVCAR8, SKOV3), lung (A549), prostate (PC3). liver (HepG2), brain (A172), and cervix (SiHa) tumor cells, hypoxia enhanced MAFF mRNA or protein expression (N: Normoxia, H: Hypoxia). *n*=3 biological replicates. Unpaired *t*-test. (g) Knockdown of *HIF1A* and *ARNT* (HIF-1β) was confirmed by qRT PCR. *n*=3 biological replicates. One-Way ANOVA with multiple comparisons. (h) Knocking down *EPAS1* (HIF-2α) using a variety of clones of shRNA did not affect MAFF induction under hypoxia measured using Western blot. Graphs represent the mean per group and error bars represent the SEM.

Supplementary Figure 3.

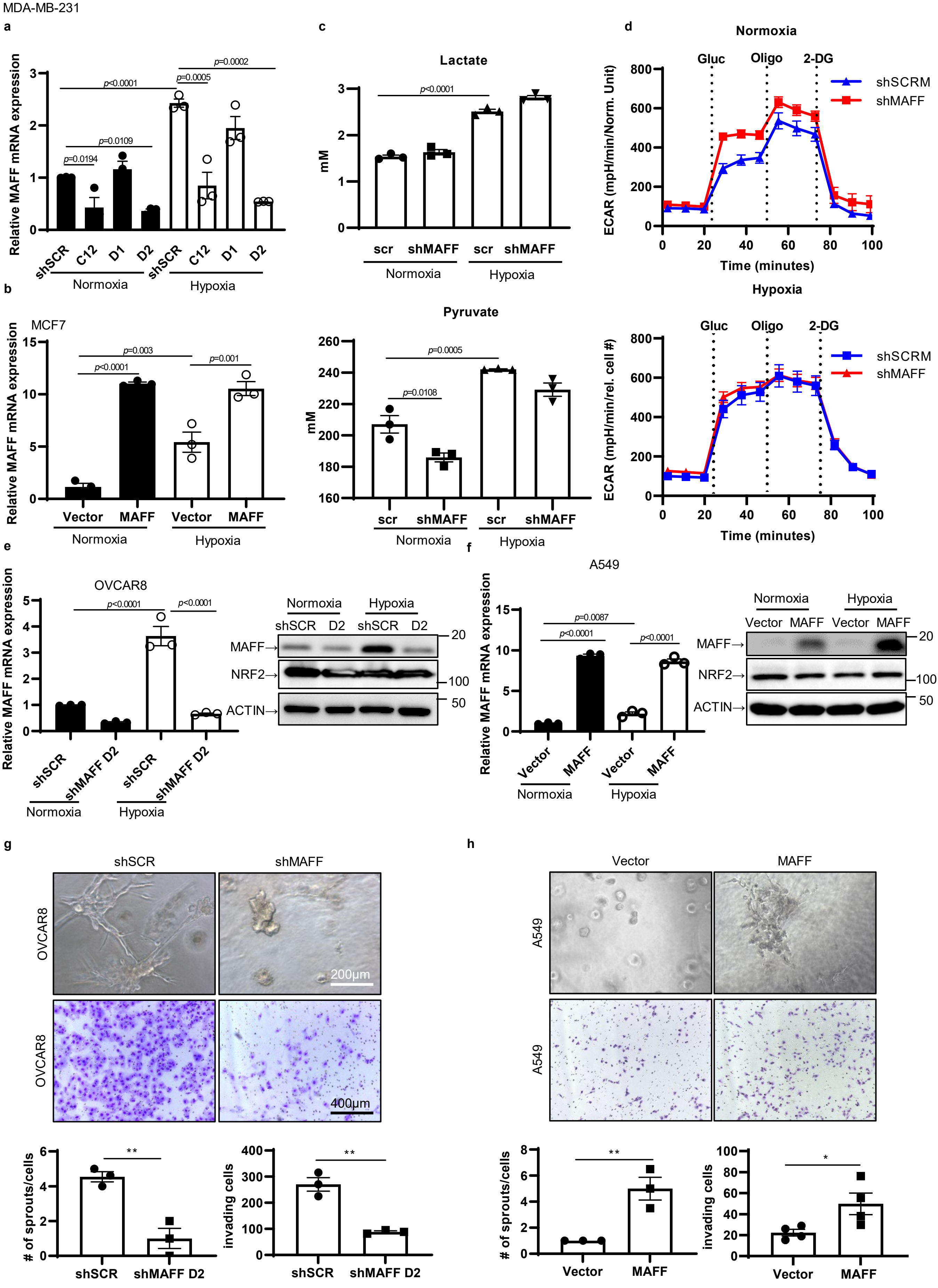

Supplementary Figure 3. MAFF regulates tumor cell invasion

MAFF was knocked down or overexpressed in MDA-MB-231 (a) or MCF7 (b) cells. mRNA expression was evaluated using qRT-PCR. *n*=3 biological replicates. One-Way ANOVA with multiple comparisons. (c) The role of MAFF in tumor metabolism was determined by measuring lactate and pyruvate levels from cell media. *n*=3 biological replicates. Student's unpaired *t*-test. (d) Seahorse FX analyzer (*Agilent*) measured extracellular acidification rates (ECAR) to compare glycolysis rates with or without MAFF knockdown. *n*=10 technical replicates. Experiments were repeated twice. (e-h) MAFF knockdown or overexpression in OVCAR8 or A549 with high NRF2 expression also changed tumor cell invasion on collagen (upper panels, *n*=3) and through Matrigel-coated transwell membranes (lower panels, *n*=4). One-Way ANOVA with multiple comparisons. Graphs represent the mean per group and error bars represent the SEM.

Supplementary Figure 4.

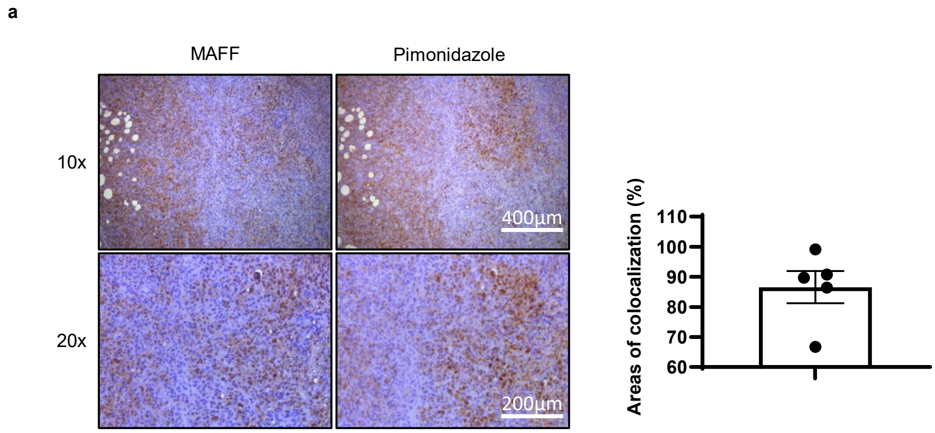

Supplementary Figure 4. MAFF regulates *in vivo* tumor metastasis

(a) Overlapping areas of MAFF expression and pimonidazole, a hypoxia marker, were observed in mouse tumor tissues (average 86.5%, n=5). Graph represents the mean, and the error bar represents the SEM.

Supplementary Figure 5.

a

Condition	Gene	Fold changes	Funcrtional Annotation
NORMOXIA	CD274	0.726438	positive regulation of cell migration
	HMGA2	1.60103	epithelial to mesenchymal transition
	MCAM	0.735603	positive regulation of cell migration
	PDGFB	1.624505	positive regulation of cell migration
	SOX9	0.617281	regulation of cell adhesion
	TGFB2	1.603251	epithelial to mesenchymal transition
	SMAD7	1.594385	negative regulation of cell migration
	COL1A1	1.638073	positive regulation of cell migration
	NEDD9	1.476314	cell adhesion
	IL11	0.629815	Wound healing
	ITGB1	0.759436	cell adhesion
	ANGPTL4	1.654047	Angiogenesis
	PGF	1.406393	Cell adhesion
	TNC	0.466516	Cell adhesion
	BMP4	1.492778	positive regulation of cell migration
	RHOB	1.976571	negative regulation of cell migration
Hypoxia	S100A4	1.558329	epithelial to mesenchymal transition
	TGFB2	1.573525	epithelial to mesenchymal transition
	COL1A1	1.605475	positive regulation of cell migration
	IL11	0.528143	Wound healing
	TNC	0.621575	Cell adhesion
	BMP4	1.400556	positive regulation of cell migration
	SRF	0.692555	Angiogenesis

b

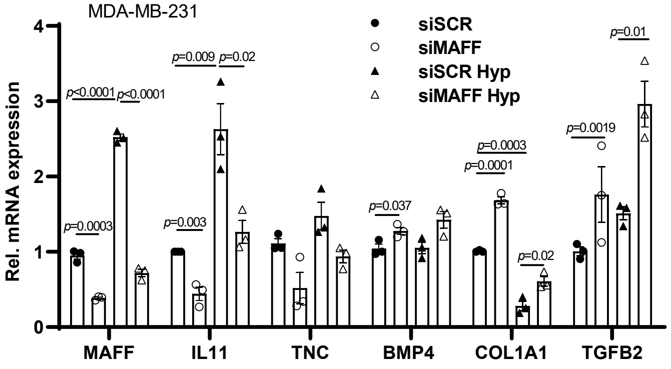

c

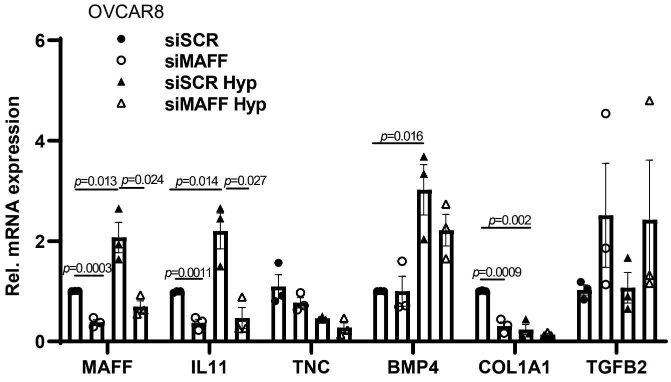

Supplementary Figure 5. MAFF target gene identification by genome wide analysis

Direct targets of MAFF were identified by ChIP sequencing using V5-MAFF overexpressing MDA-MB-231 cells with knockdown of endogenous MAFF (a) Among overlapping genes from RNA-sequencing and ChIP-sequencing, data mining by DAVID and Pubmatrix further identified 16 genes under normoxia and 7 genes under hypoxia. Among these genes, under both normoxia and hypoxia, 3 (*TGFB2, BMP4, COL1A1*) were upregulated when MAFF was knocked down, and 2 genes (*IL11, TNC*) were downregulated when MAFF was inhibited (b) Sequencing data was validated by performing qPCR in MDA-MB-231 cells. Expression of *IL11* and *TGFB2* were significantly altered by MAFF inhibition and by hypoxia. *n*=3 biological replicates. Multiple Unpaired *t*-test. (c) In OVCAR8 cells with high NRF2 expression, only *IL11* showed the consistent results with MDA-MB-231 when MAFF was knocked down or when cells were treated under hypoxia as we observed from MDA-MB-231 cells. *n*=3 biological replicates. Multiple Unpaired *t*-test. Graphs represent the mean per group and error bars represent the SEM.

Supplementary Figure 6.

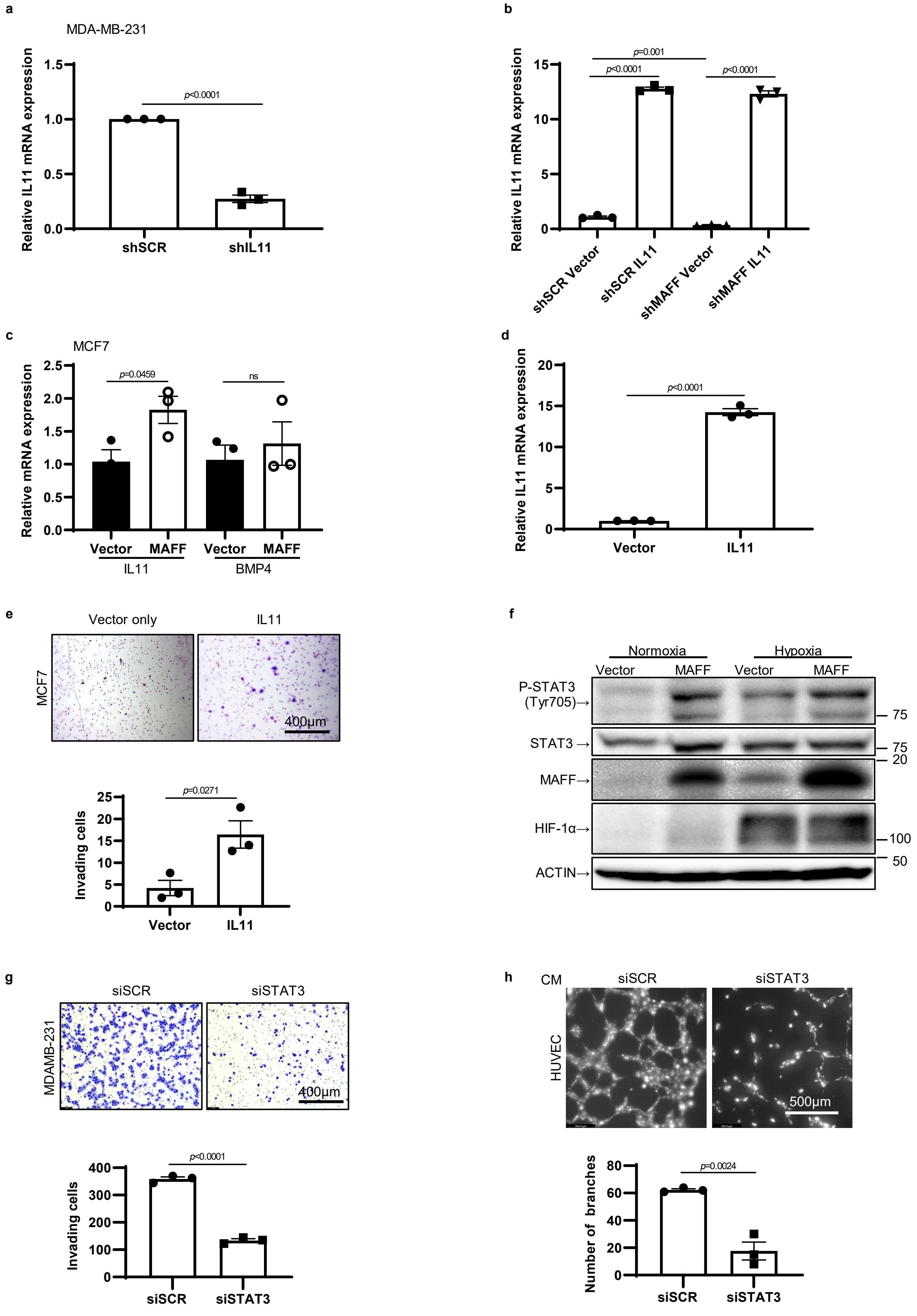

Supplementary Figure 6. MAFF-mediated IL11 regulates tumor cell invasion

(a-b) *IL11* mRNA expression was measured in MDA-MB-231 cells after *IL11* knockdown or overexpression using qRT-PCR. *n*=3 biological replicates. Unpaired *t*-test. (c) MAFF overexpression in MCF7 cells increased expression of *IL11* but not *BMP4*, which was measured by qRT-PCR. *n*=3 biological replicates. Unpaired *t*-test. (d) mRNA expression of *IL11* was confirmed after ectopic expression of IL11 by qRT-PCR. *n*=3 biological replicates. Unpaired *t*-test. (e) Overexpression of IL11 increased cell invasion of MCF7 through Matrigel coated transwell. *n*=3 biological replicates. Unpaired *t*-test. (f) Overexpression of MAFF in MCF7 cells increased phospho-STAT3. (g) Knocking down STAT3 in MDA-MB-231 using siRNA decreased tumor cell invasion through Matrigel coated transwell. *n*=3 biological replicates. Unpaired *t*-test. (h) Cell media from MDA-MB-213 with STAT3 knockdown reduced tube formation of GFP-labelled HUVEC cells on growth factor reduced matrigel (CM: Cell Media). *n*=3 biological replicates. Unpaired *t*-test. Graphs represent the mean per group and error bars represent the SEM.

Supplementary Figure 7.

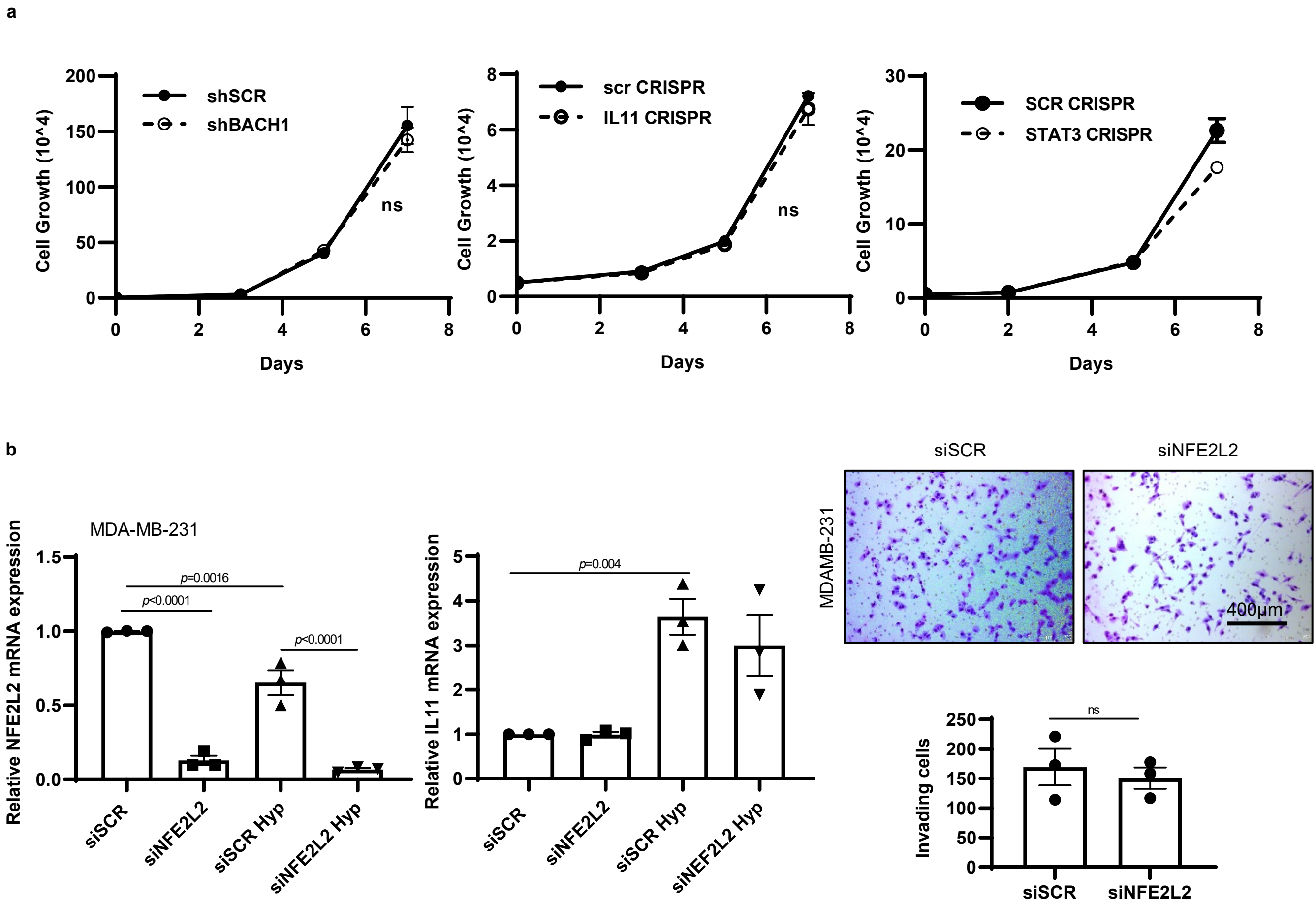

Supplementary Figure 7. MAFF binds to BACH1 to activate IL11-STAT3 pathways

(a) Inhibition of BACH1, IL11, or STAT3 did not change tumor cell growth. *n*=3 biological replicates. Unpaired *t*-test. (b) qRT-PCR data showed that knocking down *NRF2* did not change *IL11* mRNA ecrepssion in MDA-MB-231. *n*=3 biological replicates. One-Way ANOVA with multiple comparisons. (C) Knocking down *NRF2* did not alter tumor cell invasion through Matrigel coated transwells. *n*=3 biological replicates. Student's unpaired *t*-test. Graphs represent the mean per group and error bars represent the SEM.

Supplementary Figure 8.

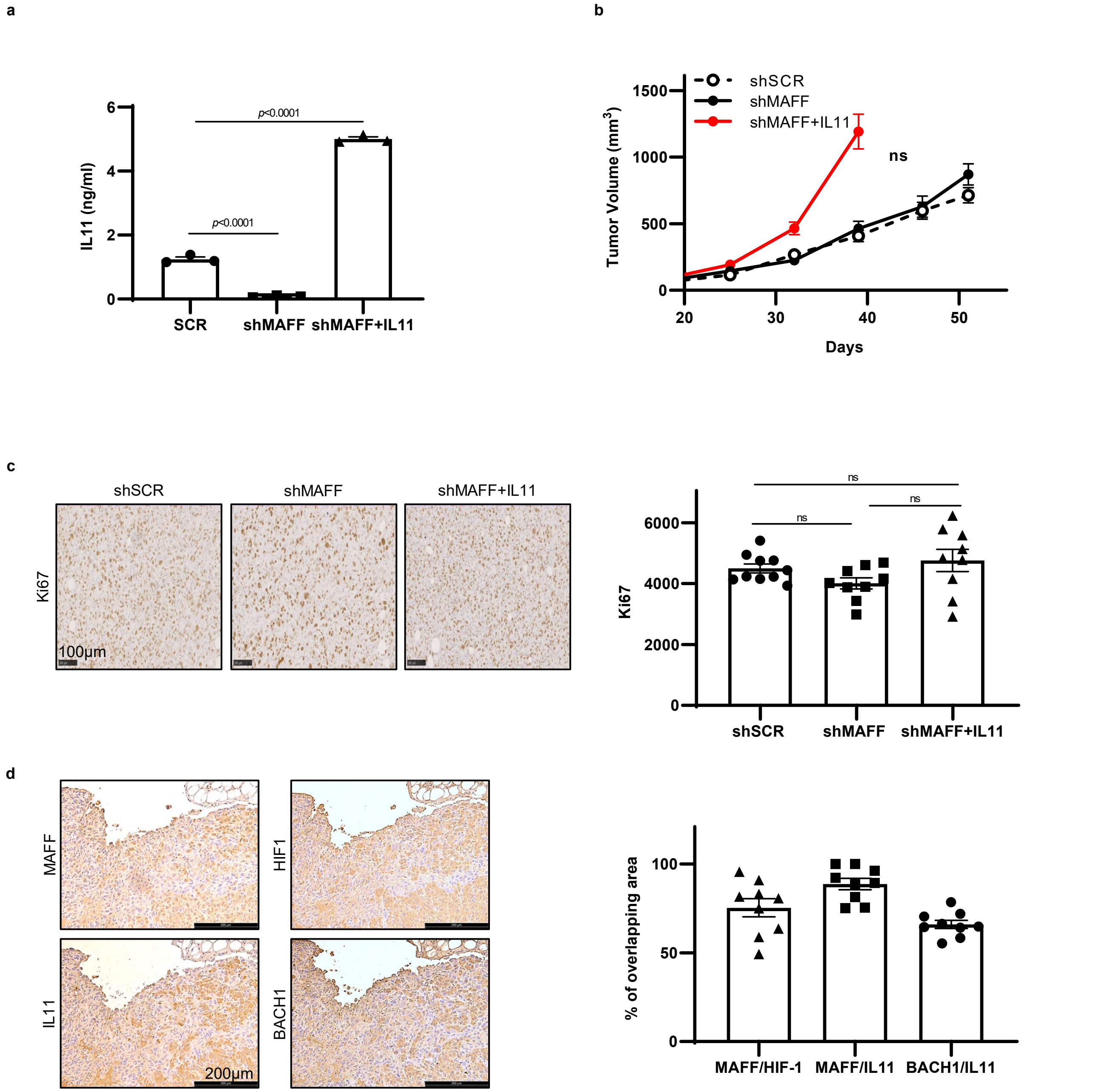

Supplementary Figure 8. MAFF enhances tumor metastasis through IL11/STAT3 pathways *in vivo* and their combined expression predicts patient survival

(a) Secreted IL11 levels were measured using ELISA before injecting MDA-MB-231 cells with or without sh*MAFF* as well as IL11 overexpression. *n*=3 biological replicates. One-Way ANOVA with multiple comparisons. (b) Growth of MDA-MB-231 injected into mammary fat pad of SCID mice were measured every week to compare the effect of MAFF and IL11 expression on tumor growth *in vivo*. *n*=10 for shSCR and shMAFF, *n*=9 for shMAFF+IL11. One-Way ANOVA. (c) Tumor cell proliferation was determined in primary tumor tissues using Ki67 staining. *n*=10 for shSCR *n*=9 for shMAFF and shMAFF+IL11. One-Way ANOVA with multiple comparisons. (d) Colocalization of MAFF and HIF-1, MAFF and IL11, and BACH1 and IL11 were determined by IHC and ImageJ analysis. *n=9*. Graphs represent the mean per group and error bars represent the SEM.
