# Supplementary material for: The HIF target MAFF promotes tumor invasion and metastasis through IL11 and STAT3 signaling
Source: Nat Commun. 2021 Jul 14;12:4308. doi: 10.1038/s41467-021-24631-6 (PMC8280233; doi:10.1038/s41467-021-24631-6)
Supplement: Supplementary file 3 — Description of Additional Supplementary Files [file 41467_2021_24631_MOESM3_ESM.docx]

**Title: Supplementary Data 1.**

Description: Top 50 hypoxia regulated genes in RCC4-VHL cells treated under hypoxia. This table contains information about top 50 hypoxia regulated genes we identified from microarray data in RCC4-VHL cells.

**Title: Supplementary Data 2.**

Description: This table contains information for MAFF regulated genes identified from both RNA-sequencing and ChIP-sequencing.

**Title: Supplementary Data 3.**

Description: Target sequences for shRNAs and Cas9-CRISPR. This table contains information for sequences of shRNAs or Cas9/CRISPR to knockdown or to knockout target genes.

**Title: Supplementary Data 4.**

Description: Primers for qPCR. This table contains information for primer sequences we used for qPCR to determine mRNA expression.

**Title: Supplementary Data 5.**

Description: Primers for ChIP. This table contains information for primer sequences we used for ChIP PCR to determine direct binding of HIF-1 or MAFF on MAFF or IL11 promoters.

**Title: Supplementary Data 6.**

Description: Promoter sequences for luciferase reporter assays. This data contains information for promoter sequences used for luciferase reporter assays to determine specific binding of HIF-1 or MAFF on target sequences.
