# Supplementary material for: The HIF target MAFF promotes tumor invasion and metastasis through IL11 and STAT3 signaling
Source: Nat Commun. 2021 Jul 14;12:4308. doi: 10.1038/s41467-021-24631-6 (PMC8280233; doi:10.1038/s41467-021-24631-6)
Supplement: Supplementary file 4 — Dataset 1 [file 41467_2021_24631_MOESM4_ESM.pptx]

## Slide 1
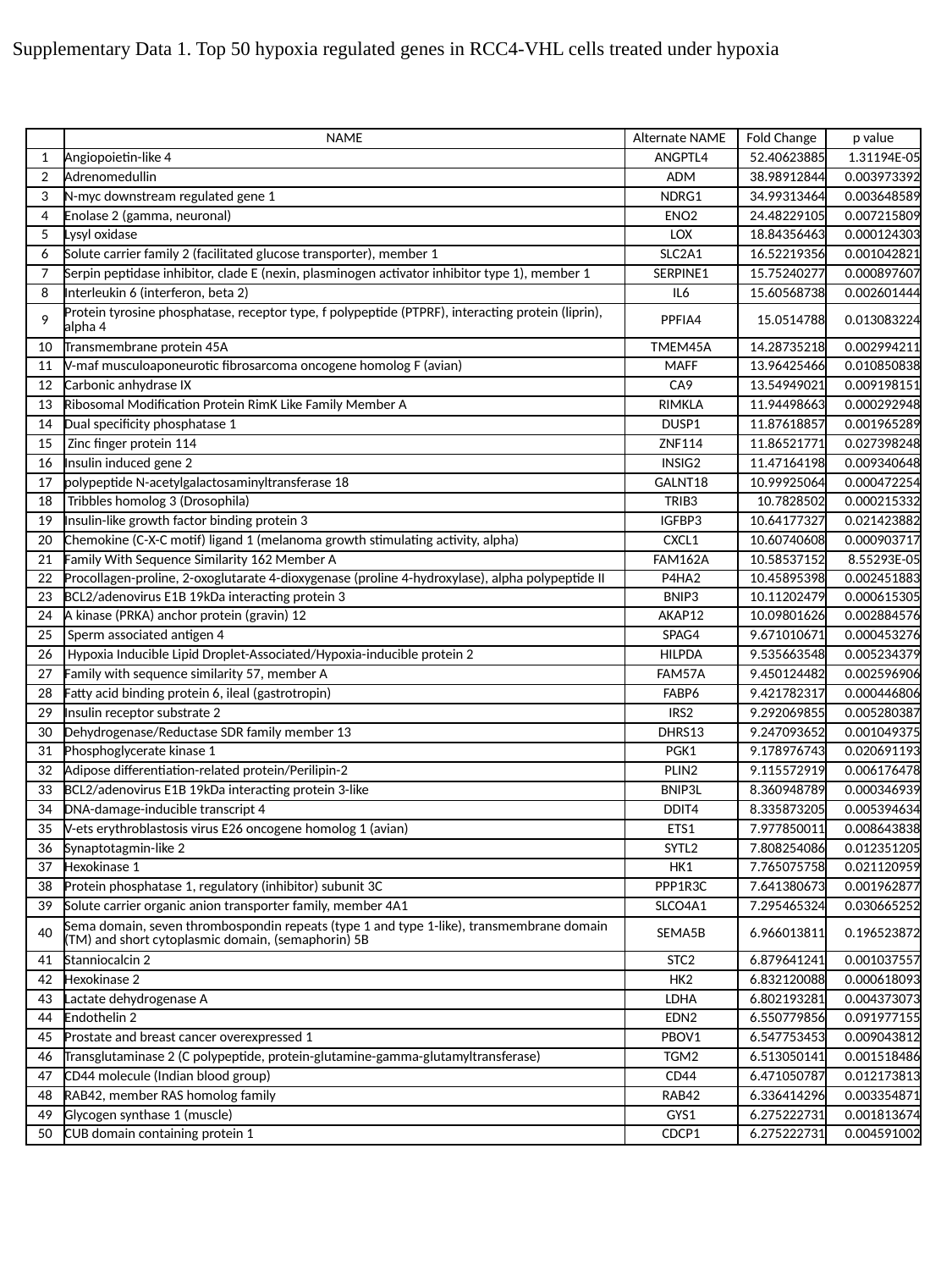

Supplementary Data 1. Top 50 hypoxia regulated genes in RCC4-VHL cells treated under hypoxia
| | NAME | Alternate NAME | Fold Change | p value |
| --- | --- | --- | --- | --- |
| 1 | Angiopoietin-like 4 | ANGPTL4 | 52.40623885 | 1.31194E-05 |
| 2 | Adrenomedullin | ADM | 38.98912844 | 0.003973392 |
| 3 | N-myc downstream regulated gene 1 | NDRG1 | 34.99313464 | 0.003648589 |
| 4 | Enolase 2 (gamma, neuronal) | ENO2 | 24.48229105 | 0.007215809 |
| 5 | Lysyl oxidase | LOX | 18.84356463 | 0.000124303 |
| 6 | Solute carrier family 2 (facilitated glucose transporter), member 1 | SLC2A1 | 16.52219356 | 0.001042821 |
| 7 | Serpin peptidase inhibitor, clade E (nexin, plasminogen activator inhibitor type 1), member 1 | SERPINE1 | 15.75240277 | 0.000897607 |
| 8 | Interleukin 6 (interferon, beta 2) | IL6 | 15.60568738 | 0.002601444 |
| 9 | Protein tyrosine phosphatase, receptor type, f polypeptide (PTPRF), interacting protein (liprin), alpha 4 | PPFIA4 | 15.0514788 | 0.013083224 |
| 10 | Transmembrane protein 45A | TMEM45A | 14.28735218 | 0.002994211 |
| 11 | V-maf musculoaponeurotic fibrosarcoma oncogene homolog F (avian) | MAFF | 13.96425466 | 0.010850838 |
| 12 | Carbonic anhydrase IX | CA9 | 13.54949021 | 0.009198151 |
| 13 | Ribosomal Modification Protein RimK Like Family Member A | RIMKLA | 11.94498663 | 0.000292948 |
| 14 | Dual specificity phosphatase 1 | DUSP1 | 11.87618857 | 0.001965289 |
| 15 | Zinc finger protein 114 | ZNF114 | 11.86521771 | 0.027398248 |
| 16 | Insulin induced gene 2 | INSIG2 | 11.47164198 | 0.009340648 |
| 17 | polypeptide N-acetylgalactosaminyltransferase 18 | GALNT18 | 10.99925064 | 0.000472254 |
| 18 | Tribbles homolog 3 (Drosophila) | TRIB3 | 10.7828502 | 0.000215332 |
| 19 | Insulin-like growth factor binding protein 3 | IGFBP3 | 10.64177327 | 0.021423882 |
| 20 | Chemokine (C-X-C motif) ligand 1 (melanoma growth stimulating activity, alpha) | CXCL1 | 10.60740608 | 0.000903717 |
| 21 | Family With Sequence Similarity 162 Member A | FAM162A | 10.58537152 | 8.55293E-05 |
| 22 | Procollagen-proline, 2-oxoglutarate 4-dioxygenase (proline 4-hydroxylase), alpha polypeptide II | P4HA2 | 10.45895398 | 0.002451883 |
| 23 | BCL2/adenovirus E1B 19kDa interacting protein 3 | BNIP3 | 10.11202479 | 0.000615305 |
| 24 | A kinase (PRKA) anchor protein (gravin) 12 | AKAP12 | 10.09801626 | 0.002884576 |
| 25 | Sperm associated antigen 4 | SPAG4 | 9.671010671 | 0.000453276 |
| 26 | Hypoxia Inducible Lipid Droplet-Associated/Hypoxia-inducible protein 2 | HILPDA | 9.535663548 | 0.005234379 |
| 27 | Family with sequence similarity 57, member A | FAM57A | 9.450124482 | 0.002596906 |
| 28 | Fatty acid binding protein 6, ileal (gastrotropin) | FABP6 | 9.421782317 | 0.000446806 |
| 29 | Insulin receptor substrate 2 | IRS2 | 9.292069855 | 0.005280387 |
| 30 | Dehydrogenase/Reductase SDR family member 13 | DHRS13 | 9.247093652 | 0.001049375 |
| 31 | Phosphoglycerate kinase 1 | PGK1 | 9.178976743 | 0.020691193 |
| 32 | Adipose differentiation-related protein/Perilipin-2 | PLIN2 | 9.115572919 | 0.006176478 |
| 33 | BCL2/adenovirus E1B 19kDa interacting protein 3-like | BNIP3L | 8.360948789 | 0.000346939 |
| 34 | DNA-damage-inducible transcript 4 | DDIT4 | 8.335873205 | 0.005394634 |
| 35 | V-ets erythroblastosis virus E26 oncogene homolog 1 (avian) | ETS1 | 7.977850011 | 0.008643838 |
| 36 | Synaptotagmin-like 2 | SYTL2 | 7.808254086 | 0.012351205 |
| 37 | Hexokinase 1 | HK1 | 7.765075758 | 0.021120959 |
| 38 | Protein phosphatase 1, regulatory (inhibitor) subunit 3C | PPP1R3C | 7.641380673 | 0.001962877 |
| 39 | Solute carrier organic anion transporter family, member 4A1 | SLCO4A1 | 7.295465324 | 0.030665252 |
| 40 | Sema domain, seven thrombospondin repeats (type 1 and type 1-like), transmembrane domain (TM) and short cytoplasmic domain, (semaphorin) 5B | SEMA5B | 6.966013811 | 0.196523872 |
| 41 | Stanniocalcin 2 | STC2 | 6.879641241 | 0.001037557 |
| 42 | Hexokinase 2 | HK2 | 6.832120088 | 0.000618093 |
| 43 | Lactate dehydrogenase A | LDHA | 6.802193281 | 0.004373073 |
| 44 | Endothelin 2 | EDN2 | 6.550779856 | 0.091977155 |
| 45 | Prostate and breast cancer overexpressed 1 | PBOV1 | 6.547753453 | 0.009043812 |
| 46 | Transglutaminase 2 (C polypeptide, protein-glutamine-gamma-glutamyltransferase) | TGM2 | 6.513050141 | 0.001518486 |
| 47 | CD44 molecule (Indian blood group) | CD44 | 6.471050787 | 0.012173813 |
| 48 | RAB42, member RAS homolog family | RAB42 | 6.336414296 | 0.003354871 |
| 49 | Glycogen synthase 1 (muscle) | GYS1 | 6.275222731 | 0.001813674 |
| 50 | CUB domain containing protein 1 | CDCP1 | 6.275222731 | 0.004591002 |
