# Supplementary material for: The HIF target MAFF promotes tumor invasion and metastasis through IL11 and STAT3 signaling
Source: Nat Commun. 2021 Jul 14;12:4308. doi: 10.1038/s41467-021-24631-6 (PMC8280233; doi:10.1038/s41467-021-24631-6)
Supplement: Supplementary file 6 — Dataset 3 [file 41467_2021_24631_MOESM6_ESM.pptx]

## Slide 1
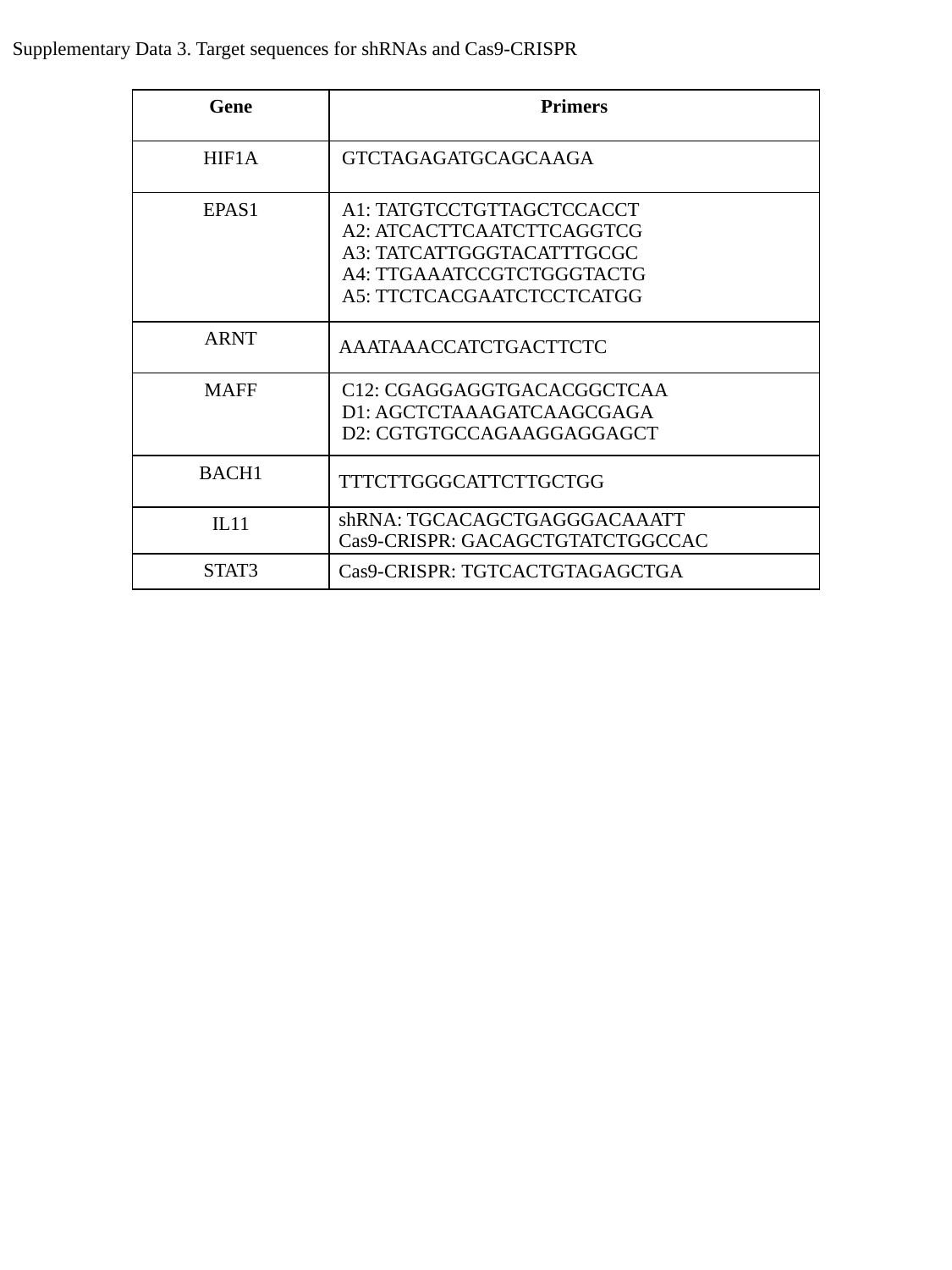

Supplementary Data 3. Target sequences for shRNAs and Cas9-CRISPR
| Gene | Primers |
| --- | --- |
| HIF1A | GTCTAGAGATGCAGCAAGA |
| EPAS1 | A1: TATGTCCTGTTAGCTCCACCT A2: ATCACTTCAATCTTCAGGTCG A3: TATCATTGGGTACATTTGCGC A4: TTGAAATCCGTCTGGGTACTG A5: TTCTCACGAATCTCCTCATGG |
| ARNT | AAATAAACCATCTGACTTCTC |
| MAFF | C12: CGAGGAGGTGACACGGCTCAA D1: AGCTCTAAAGATCAAGCGAGA D2: CGTGTGCCAGAAGGAGGAGCT |
| BACH1 | TTTCTTGGGCATTCTTGCTGG |
| IL11 | shRNA: TGCACAGCTGAGGGACAAATT Cas9-CRISPR: GACAGCTGTATCTGGCCAC |
| STAT3 | Cas9-CRISPR: TGTCACTGTAGAGCTGA |
