# Supplementary material for: The HIF target MAFF promotes tumor invasion and metastasis through IL11 and STAT3 signaling
Source: Nat Commun. 2021 Jul 14;12:4308. doi: 10.1038/s41467-021-24631-6 (PMC8280233; doi:10.1038/s41467-021-24631-6)
Supplement: Supplementary file 7 — Dataset 4 [file 41467_2021_24631_MOESM7_ESM.pptx]

## Slide 1
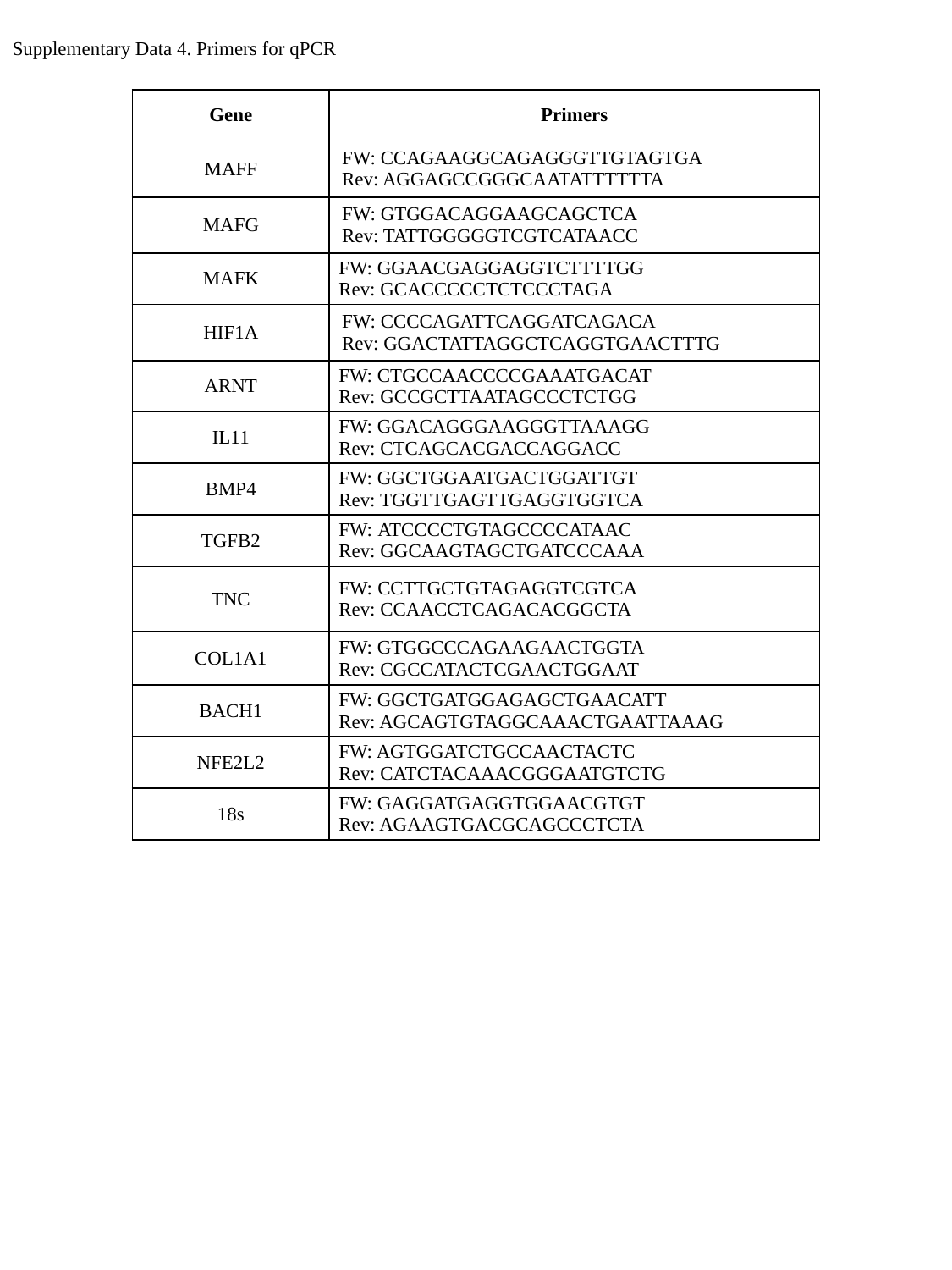

Supplementary Data 4. Primers for qPCR
| Gene | Primers |
| --- | --- |
| MAFF | FW: CCAGAAGGCAGAGGGTTGTAGTGA Rev: AGGAGCCGGGCAATATTTTTTA |
| MAFG | FW: GTGGACAGGAAGCAGCTCA Rev: TATTGGGGGTCGTCATAACC |
| MAFK | FW: GGAACGAGGAGGTCTTTTGG Rev: GCACCCCCTCTCCCTAGA |
| HIF1A | FW: CCCCAGATTCAGGATCAGACA Rev: GGACTATTAGGCTCAGGTGAACTTTG |
| ARNT | FW: CTGCCAACCCCGAAATGACAT Rev: GCCGCTTAATAGCCCTCTGG |
| IL11 | FW: GGACAGGGAAGGGTTAAAGG Rev: CTCAGCACGACCAGGACC |
| BMP4 | FW: GGCTGGAATGACTGGATTGT Rev: TGGTTGAGTTGAGGTGGTCA |
| TGFB2 | FW: ATCCCCTGTAGCCCCATAAC Rev: GGCAAGTAGCTGATCCCAAA |
| TNC | FW: CCTTGCTGTAGAGGTCGTCA Rev: CCAACCTCAGACACGGCTA |
| COL1A1 | FW: GTGGCCCAGAAGAACTGGTA Rev: CGCCATACTCGAACTGGAAT |
| BACH1 | FW: GGCTGATGGAGAGCTGAACATT Rev: AGCAGTGTAGGCAAACTGAATTAAAG |
| NFE2L2 | FW: AGTGGATCTGCCAACTACTC Rev: CATCTACAAACGGGAATGTCTG |
| 18s | FW: GAGGATGAGGTGGAACGTGT Rev: AGAAGTGACGCAGCCCTCTA |
