# Supplementary material for: The HIF target MAFF promotes tumor invasion and metastasis through IL11 and STAT3 signaling
Source: Nat Commun. 2021 Jul 14;12:4308. doi: 10.1038/s41467-021-24631-6 (PMC8280233; doi:10.1038/s41467-021-24631-6)
Supplement: Supplementary file 8 — Dataset 5 [file 41467_2021_24631_MOESM8_ESM.pptx]

## Slide 1
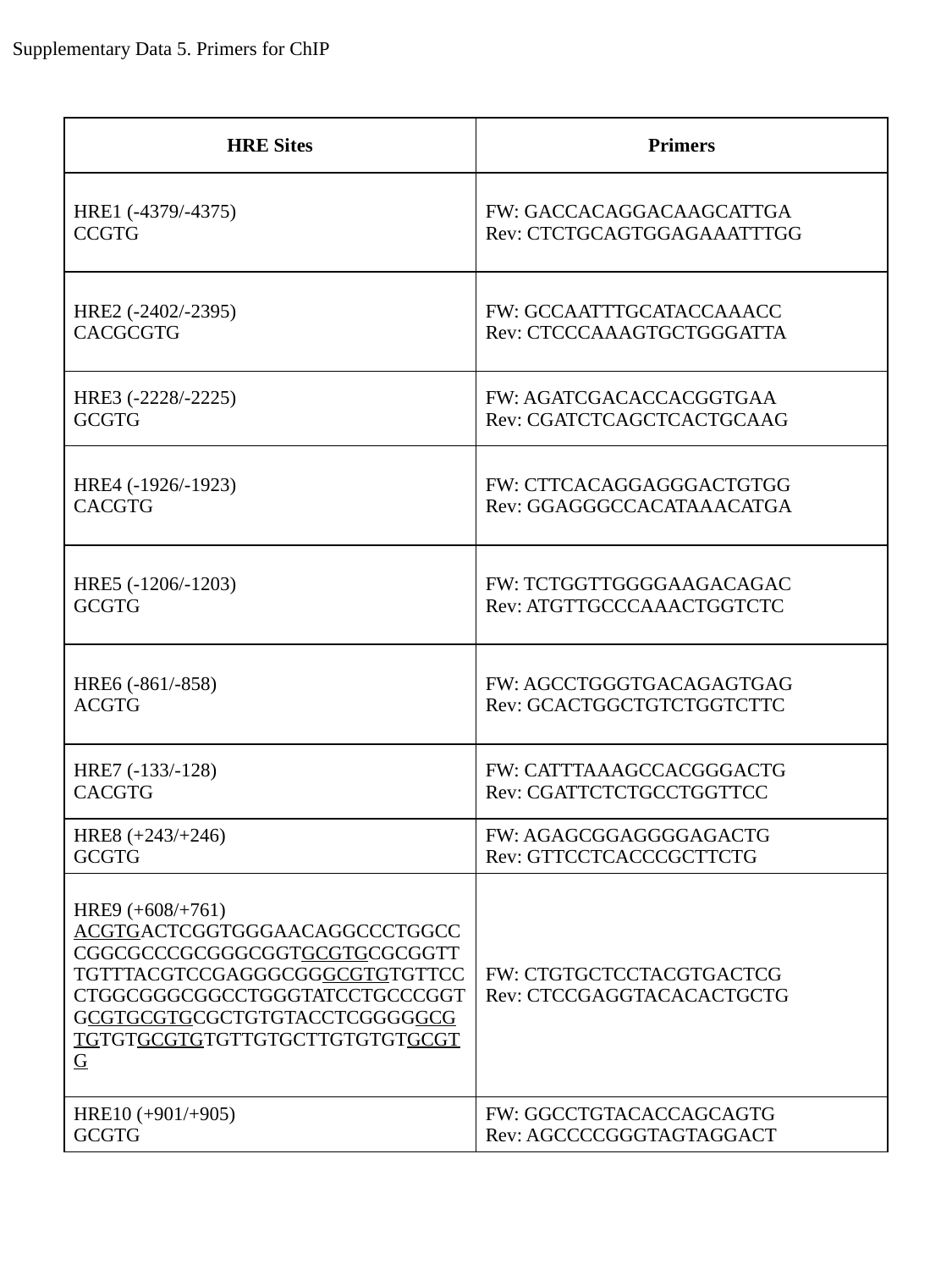

Supplementary Data 5. Primers for ChIP
| HRE Sites | Primers |
| --- | --- |
| HRE1 (-4379/-4375) CCGTG | FW: GACCACAGGACAAGCATTGA Rev: CTCTGCAGTGGAGAAATTTGG |
| HRE2 (-2402/-2395) CACGCGTG | FW: GCCAATTTGCATACCAAACC Rev: CTCCCAAAGTGCTGGGATTA |
| HRE3 (-2228/-2225) GCGTG | FW: AGATCGACACCACGGTGAA Rev: CGATCTCAGCTCACTGCAAG |
| HRE4 (-1926/-1923) CACGTG | FW: CTTCACAGGAGGGACTGTGG Rev: GGAGGGCCACATAAACATGA |
| HRE5 (-1206/-1203) GCGTG | FW: TCTGGTTGGGGAAGACAGAC Rev: ATGTTGCCCAAACTGGTCTC |
| HRE6 (-861/-858) ACGTG | FW: AGCCTGGGTGACAGAGTGAG Rev: GCACTGGCTGTCTGGTCTTC |
| HRE7 (-133/-128) CACGTG | FW: CATTTAAAGCCACGGGACTG Rev: CGATTCTCTGCCTGGTTCC |
| HRE8 (+243/+246) GCGTG | FW: AGAGCGGAGGGGAGACTG Rev: GTTCCTCACCCGCTTCTG |
| HRE9 (+608/+761) ACGTGACTCGGTGGGAACAGGCCCTGGCCCGGCGCCCGCGGGCGGTGCGTGCGCGGTTTGTTTACGTCCGAGGGCGGGCGTGTGTTCCCTGGCGGGCGGCCTGGGTATCCTGCCCGGTGCGTGCGTGCGCTGTGTACCTCGGGGGCGTGTGTGCGTGTGTTGTGCTTGTGTGTGCGTG | FW: CTGTGCTCCTACGTGACTCG Rev: CTCCGAGGTACACACTGCTG |
| HRE10 (+901/+905) GCGTG | FW: GGCCTGTACACCAGCAGTG Rev: AGCCCCGGGTAGTAGGACT |
