# Supplementary material for: The HIF target MAFF promotes tumor invasion and metastasis through IL11 and STAT3 signaling
Source: Nat Commun. 2021 Jul 14;12:4308. doi: 10.1038/s41467-021-24631-6 (PMC8280233; doi:10.1038/s41467-021-24631-6)
Supplement: Supplementary file 9 — Dataset 6 [file 41467_2021_24631_MOESM9_ESM.pptx]

## Slide 1
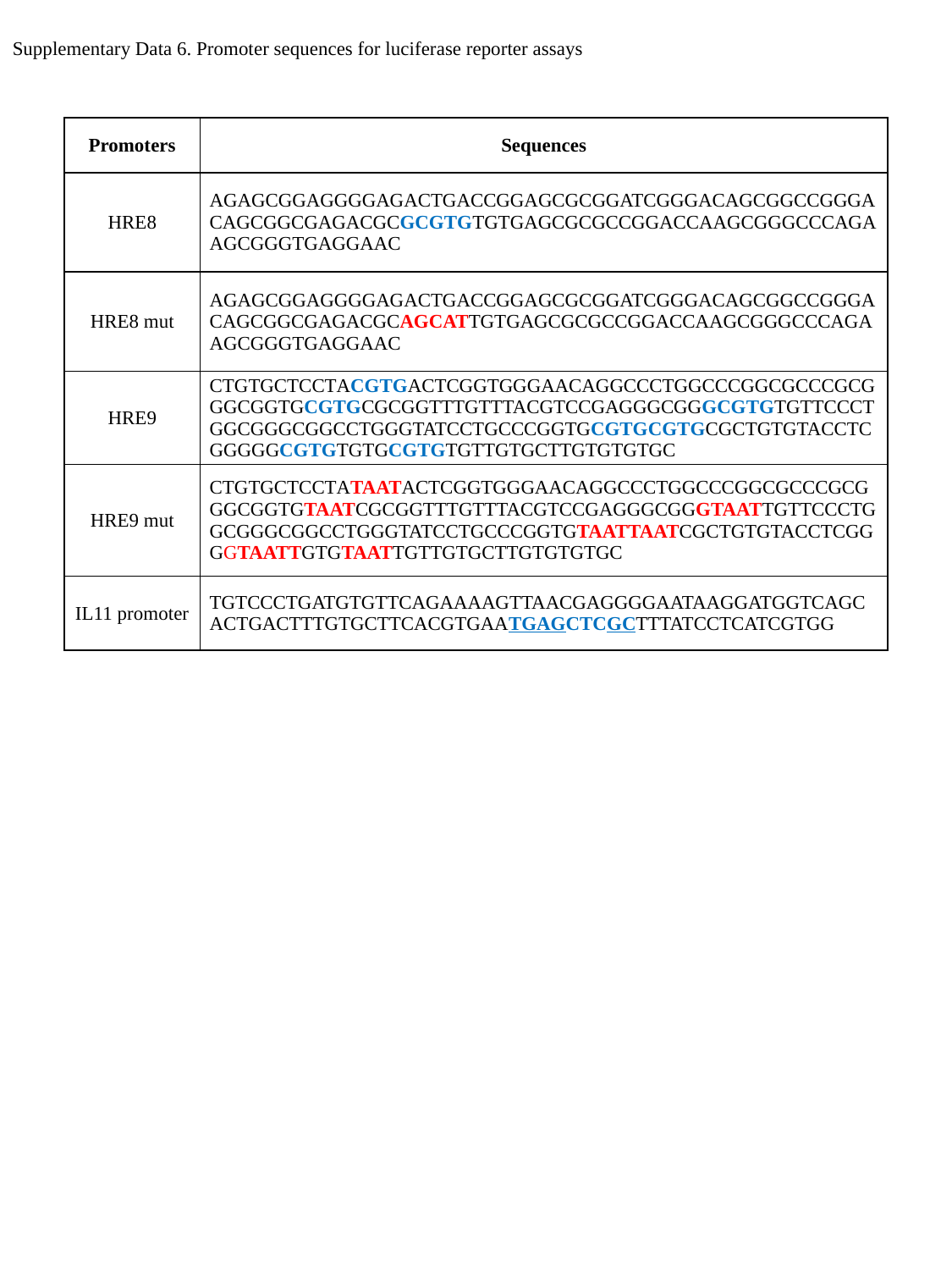

Supplementary Data 6. Promoter sequences for luciferase reporter assays
| Promoters | Sequences |
| --- | --- |
| HRE8 | AGAGCGGAGGGGAGACTGACCGGAGCGCGGATCGGGACAGCGGCCGGGACAGCGGCGAGACGCGCGTGTGTGAGCGCGCCGGACCAAGCGGGCCCAGAAGCGGGTGAGGAAC |
| HRE8 mut | AGAGCGGAGGGGAGACTGACCGGAGCGCGGATCGGGACAGCGGCCGGGACAGCGGCGAGACGCAGCATTGTGAGCGCGCCGGACCAAGCGGGCCCAGAAGCGGGTGAGGAAC |
| HRE9 | CTGTGCTCCTACGTGACTCGGTGGGAACAGGCCCTGGCCCGGCGCCCGCGGGCGGTGCGTGCGCGGTTTGTTTACGTCCGAGGGCGGGCGTGTGTTCCCTGGCGGGCGGCCTGGGTATCCTGCCCGGTGCGTGCGTGCGCTGTGTACCTCGGGGGCGTGTGTGCGTGTGTTGTGCTTGTGTGTGC |
| HRE9 mut | CTGTGCTCCTATAATACTCGGTGGGAACAGGCCCTGGCCCGGCGCCCGCGGGCGGTGTAATCGCGGTTTGTTTACGTCCGAGGGCGGGTAATTGTTCCCTGGCGGGCGGCCTGGGTATCCTGCCCGGTGTAATTAATCGCTGTGTACCTCGGGGTAATTGTGTAATTGTTGTGCTTGTGTGTGC |
| IL11 promoter | TGTCCCTGATGTGTTCAGAAAAGTTAACGAGGGGAATAAGGATGGTCAGCACTGACTTTGTGCTTCACGTGAATGAGCTCGCTTTATCCTCATCGTGG |
