# Supplementary material for: The HIF target MAFF promotes tumor invasion and metastasis through IL11 and STAT3 signaling
Source: Nat Commun. 2021 Jul 14;12:4308. doi: 10.1038/s41467-021-24631-6 (PMC8280233; doi:10.1038/s41467-021-24631-6)
Supplement: Supplementary file 10 — Reporting Summary [file 41467_2021_24631_MOESM10_ESM.pdf]

nature research

Corresponding author(s): Amato Giaccia

Last updated by author(s): March/03/2021

Reporting Summary

Nature Research wishes to improve the reproducibility of the work that we publish. This form provides structure for consistency and transparency in reporting. For further information on Nature Research policies, see Authors & Referees and the Editorial Policy Checklist .

Statistics

For all statistical analyses, confirm that the following items are present in the figure legend, table legend, main text, or Methods section.

n/a	Confirmed	
☐	☒	The exact sample size (n) for each experimental group/condition, given as a discrete number and unit of measurement
☐	☒	A statement on whether measurements were taken from distinct samples or whether the same sample was measured repeatedly
☐	☒	The statistical test(s) used AND whether they are one- or two-sided<br>*Only common tests should be described solely by name; describe more complex techniques in the Methods section.*
☒	☐	A description of all covariates tested
☒	☐	A description of any assumptions or corrections, such as tests of normality and adjustment for multiple comparisons
☐	☒	A full description of the statistical parameters including central tendency (e.g. means) or other basic estimates (e.g. regression coefficient) AND variation (e.g. standard deviation) or associated estimates of uncertainty (e.g. confidence intervals)
☐	☒	For null hypothesis testing, the test statistic (e.g. F, t, r) with confidence intervals, effect sizes, degrees of freedom and P value noted<br>*Give P values as exact values whenever suitable.*
☒	☐	For Bayesian analysis, information on the choice of priors and Markov chain Monte Carlo settings
☒	☐	For hierarchical and complex designs, identification of the appropriate level for tests and full reporting of outcomes
☐	☒	Estimates of effect sizes (e.g. Cohen's d, Pearson's r), indicating how they were calculated

Our web collection on statistics for biologists contains articles on many of the points above.

Software and code

Policy information about availability of computer code

Data collection	Leica Application Suite X (LAS X) was used to take images using Leica DMi8. Image Lab 4.0 was used to collect western blot images from Chemidoc.
Data analysis	ImageJ was used to perform image analysis for invasion assay, H&E staining, and IHC. Graphpad prism 8 was used to generate graph and to perform statistical test.

For manuscripts utilizing custom algorithms or software that are central to the research but not yet described in published literature, software must be made available to editors/reviewers. We strongly encourage code deposition in a community repository (e.g. GitHub). See the Nature Research guidelines for submitting code & software for further information.

Data

Policy information about availability of data

All manuscripts must include a data availability statement. This statement should provide the following information, where applicable:

- Accession codes, unique identifiers, or web links for publicly available datasets
- A list of figures that have associated raw data
- A description of any restrictions on data availability

Raw and processed data for RNA and ChIP-sequencing data (Fig. 5, Supplementary Fig. 5) are available in the Gene Expression Omnimus (GEO) database (http://www.ncbi.nlm.niv.gov/gds) under the accession number GSE144964. Raw data for western blotting are included in Data source. cbioportal was used to determine genetic alterations in hypoxia regulated genes. ProgGene V2, Kaplan Meier plotter (kmplot.com), and GEO datasets (GSE42568), were used to evaluate patient survival.

Field-specific reporting

Please select the one below that is the best fit for your research. If you are not sure, read the appropriate sections before making your selection.

- [x] Life sciences
- [ ] Behavioural & social sciences
- [ ] Ecological, evolutionary & environmental sciences

For a reference copy of the document with all sections, see nature.com/documents/nr-reporting-summary-flat.pdf

Life sciences study design

All studies must disclose on these points even when the disclosure is negative.

Sample size

For in vitro studies, n=3 or 4 were chosen to test statistical significance, while n=5-10 were used for in vivo studies. The number of replicates were determined based on minimum number of replicate to achieve statistical power.

Data exclusions

No data were excluded.

Replication

All attempts to replicate results were successful. esiRNA screening for cell invasion and survival was performed three times. Real time qPCR, ChIP, luciferase reporter assays were performed three times with 3 technical replicates. For in vitro invasion assay and in vivo IHC, images were taken three representative fields and averaged quantitation was used in each data point. Rest of experiments were performed with three biological replicates.

Randomization

Randomization was not required, since we did not compare any treatment groups requiring randomization to form groups.

Blinding

Quantification of in vitro invasion assay and in vivo H&E and IHC images were performed in blind ways. Rest of experiments did not require blinding for analysis.

Reporting for specific materials, systems and methods

We require information from authors about some types of materials, experimental systems and methods used in many studies. Here, indicate whether each material, system or method listed is relevant to your study. If you are not sure if a list item applies to your research, read the appropriate section before selecting a response.

Materials & experimental systems

n/a	Involved in the study	
	x	Antibodies
	x	Eukaryotic cell lines
x		Palaeontology
	x	Animals and other organisms
x		Human research participants
x		Clinical data

Methods

n/a	Involved in the study	
	x	ChIP-seq
x		Flow cytometry
x		MRI-based neuroimaging

Antibodies

Antibodies used

Western Blot: MAFF (1:1000, MilliporeSigma #M8194), MAFG (1:1000, Genetex #114541), MAFK (1:1000, R&D #MAB3809), HIF-1α (1:250, BD Transduction LaboratoriesTM #610959), HIF-2α (1:1000, Novus #100-122), HIF-1β (1:1000, BD Transduction LaboratoriesTM #611079), BACH1 (1:1000, Genetex #GTX63193), NRF2 (1:1000, Genetex GTX61763), phospho-STAT3 (Tyr705) (1:1000, Cell Signaling #9145), STAT3 (1:1000, Cell Signaling #9139), ACTIN (1:5000, Santa Cruz #sc-47778 HRP), anti-rabbit HRP antibody (1:2000, Jackson Immunoresearch #711-035-152), anti-mouse HRP antibody (1:2000, Jackson Immunoresearch #711-035-003), anti-goat HRP (1:2000, Santa Cruz #sc-2354)

Co-immunoprecipitation: MAFF: 15μg, MilliporeSigma #M8194, BACH1: 5μg, Santa Cruz #sc-14700, or IgG: Santa Cruz #sc-2027

Immunohistochemistry: MAFF (1:200, Genetex #GTX120264), BACH1 (10μg/ml, R&D #AF5776), IL11 (1:200, Genetex #GTX66720), HIF-1α (1:200, Abcam #ab2185), phospho-STAT3 (1:200, Cell Signaling #9145), MECA-32 (1:200, BD Transduction #553849), Ki67 (1:200, Thermo Scientific), monoclonal antibody for pimonidazole (1:50 Hypoxyprobe MAb 1), Biotylated anti-Rabbit (1:200, Vector Laboratories #BA-1000), Biotylated anti-mouse (1:200, Vector Laboratories #BA-9200), Biotylated anti-rat (1:200, Vector Laboratories #BA-9400)

ChIP: MAFF: MilliporeSigma #M8194, V5: Cell Signaling #13202, rabbit IgG (Santa Cruz #SC-2027)

Validation

MAFF, MAFG, and MAFK antibodies were validated by knocking down each of these genes (Supplementary Fig. 2a). All other antibodies were used based on manufacturer's protocols, which confirm now cross-reactivity between species.

Eukaryotic cell lines

Policy information about cell lines

Cell line source(s)	MDA-MB-231, MCF7, A549, PC3, HCT116, HepG2, A172, SiHa: ATCC<br>OVCAR8: National Cancer Institute-Frederick DCTD tumor cell line repository<br>SKOV3, and SKOV3i.p were obtained from Dr. Erinn Rankin's laboratory (Stanford University), which was originally gifted from Dr. Gordon Mills in MD Anderson Cancer Center..
Authentication	MDA-MB-231 and HepG2 were directly purchased from ATCC. MCF7 was authenticated from ATCC. Rest of cell lines were used without authentication.
Mycoplasma contamination	All cells were mycoplasma free when tested with MycoAlert mycoplamsa detection kit purchased from Lonza.
Commonly misidentified lines (See ICLAC register)	Commonly misidentified cell lines were not used in this study.

Animals and other organisms

Policy information about studies involving animals; ARRIVE guidelines recommended for reporting animal research

Laboratory animals	Female athymic nude mice (Crl:NU(NCr)-Foxn1nu, Charles River) and NSG mice (NOD.Cg-PrkdcscidIl2rgtmlWjl/Szj, Jackson Laboratory #005557 at 6-8 weeks old were used. Animals were housed at room temperature, under 40-70% humidity, with a 12 hour light/12 hour dark schedule. Mice were fed with dry granule food and had free access to reverse osmosis water.
Wild animals	The study did not involve wild animals.
Field-collected samples	The study did not involve samples collected from the field.
Ethics oversight	All animal studies were conducted with approval by the Institutional Animal Care and Use Committee (IACUC) at Stanford University.

Note that full information on the approval of the study protocol must also be provided in the manuscript.

ChIP-seq

Data deposition

- [x] Confirm that both raw and final processed data have been deposited in a public database such as GEO.
- [x] Confirm that you have deposited or provided access to graph files (e.g. BED files) for the called peaks.

Data access links<br>*May remain private before publication.*	https://www.ncbi.nlm.nih.gov/geo/query/acc.cgi?acc=GSE144964
Files in database submission	MAFF-V5 ChIP sequencing, MAFF RNA sequencing under normoxia and hypoxia (raw and processed)
Genome browser session (e.g. UCSC)	No longer applicable.

Methodology

Replicates	RNA sequnecing with three replicates for each condition, ChIP sequencing with two replicates
Sequencing depth	50 bp, single end reading for RNA seq, 150 bp, paired end reading for ChIP seq
Antibodies	V5 (Cell Signaling #13202)
Peak calling parameters	Peak calling was performed using MACS2 with settings for peak enrichment >20, peak-to-background enrichment >3
Data quality	For Peak calling, we used the setting of a minimum ratio between uniquely and repetitively mapped reads of 3:1, a kernel bandwith of 30.0, which enabled FDR calculation. MAFF-bound sites with a false discovery rate (FDR) of 5% were used for further analysis.
Software	Peak calling was performed using MACS2 with settings for peak enrichment >20, peak-to-background enrichment >3, a minimum ratio between uniquely and repetitively mapped reads of 3:1, a kernel bandwith of 30.0, and enabling FDR calculation. Binding motif was acquired using MEME analysis.
