# Supplementary material for: The HIF target MAFF promotes tumor invasion and metastasis through IL11 and STAT3 signaling
Source: Nat Commun. 2021 Jul 14;12:4308. doi: 10.1038/s41467-021-24631-6 (PMC8280233; doi:10.1038/s41467-021-24631-6)

Figure 2b

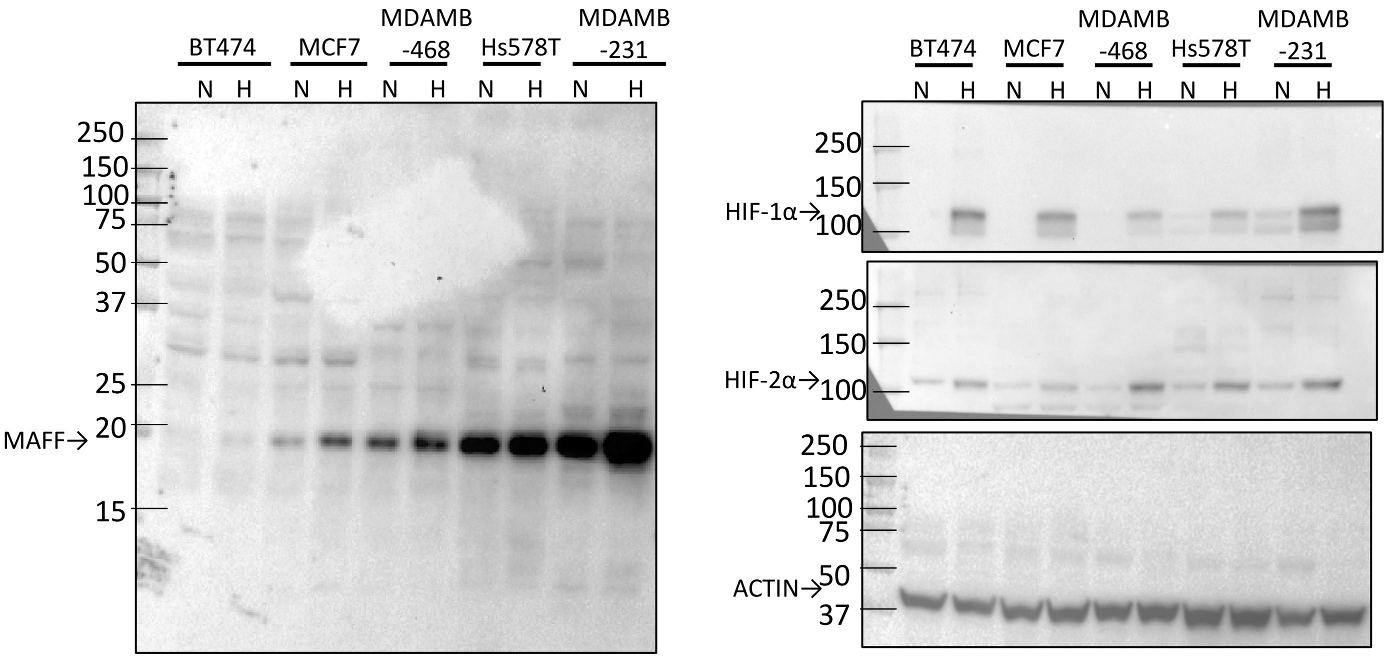

Figure 2d

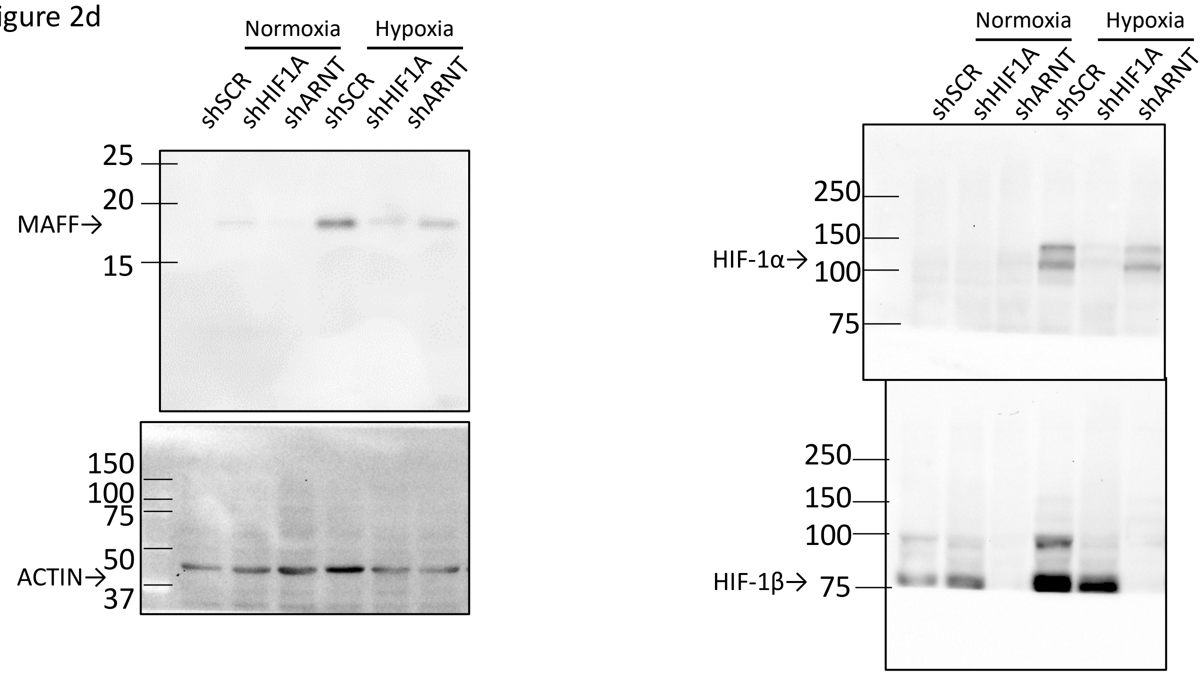

Figure 3a

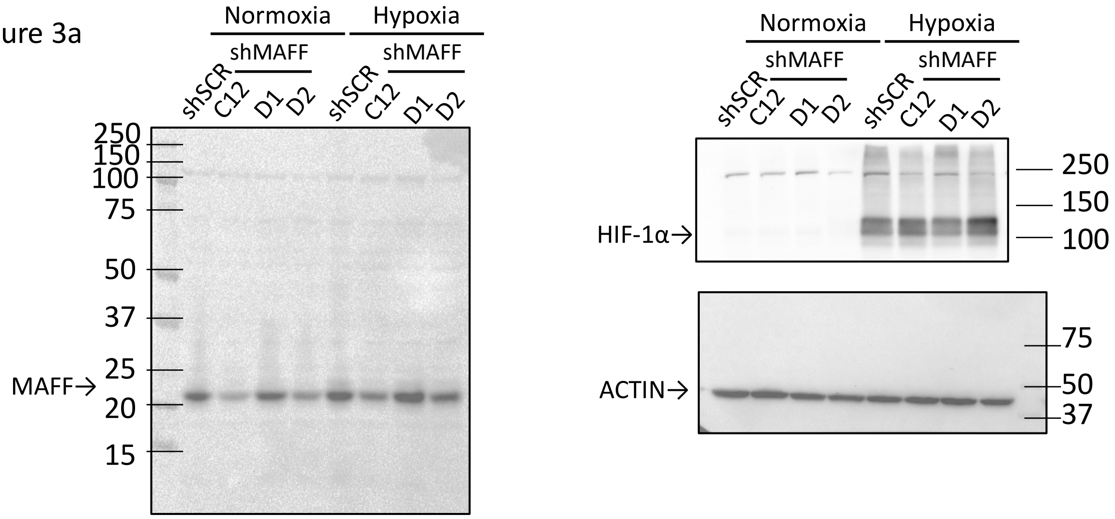

Figure 3d

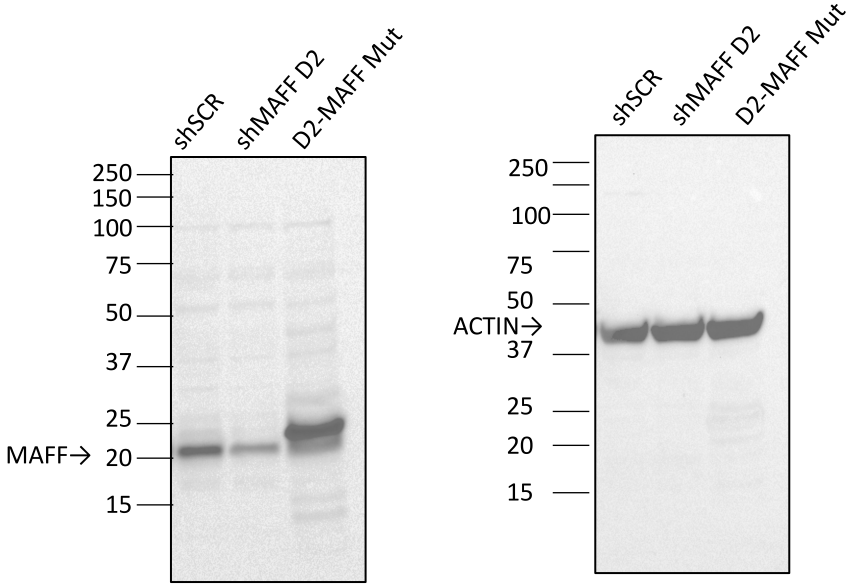

Figure 3e

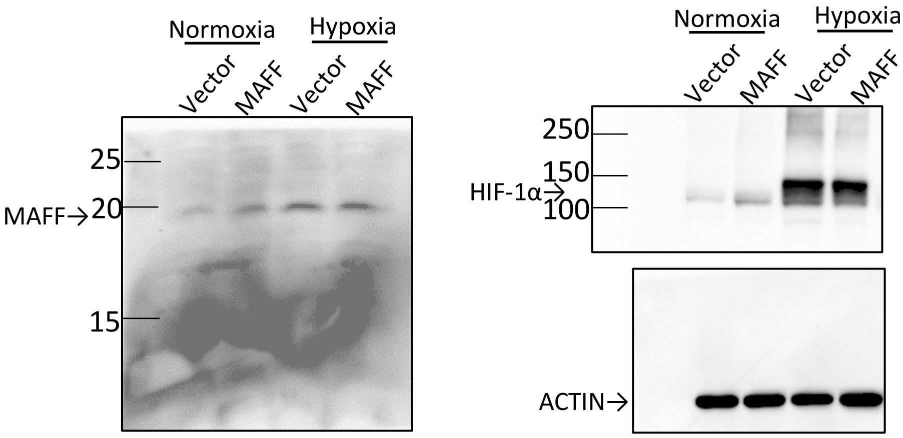

Figure 6c

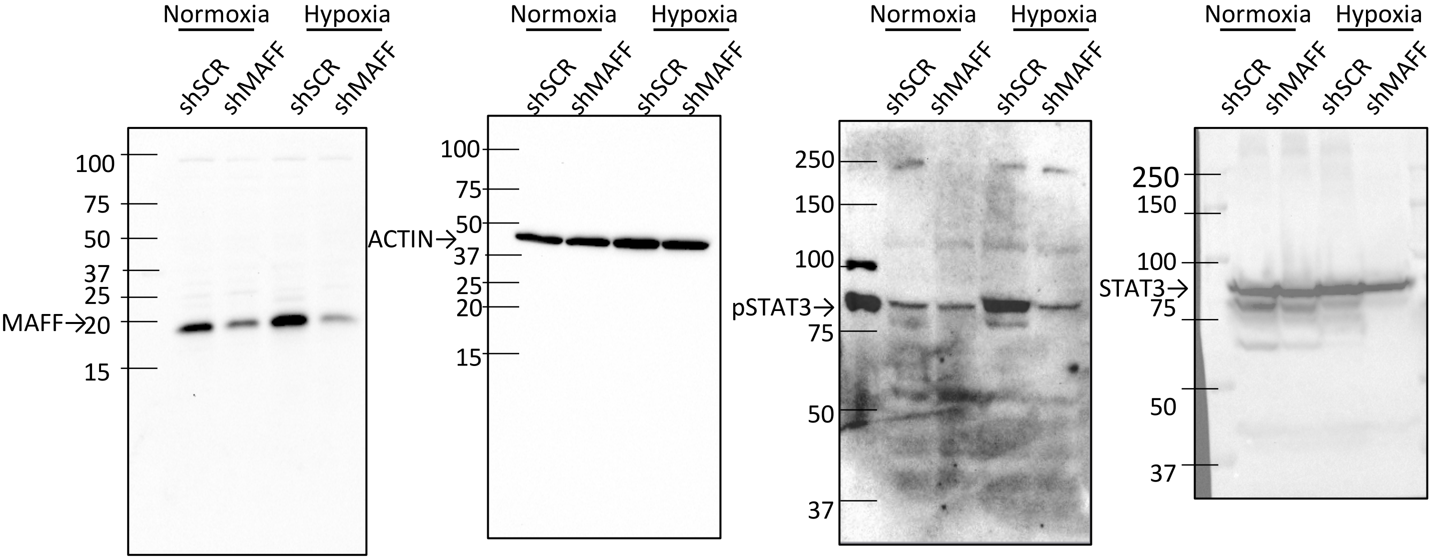

Figure 6d

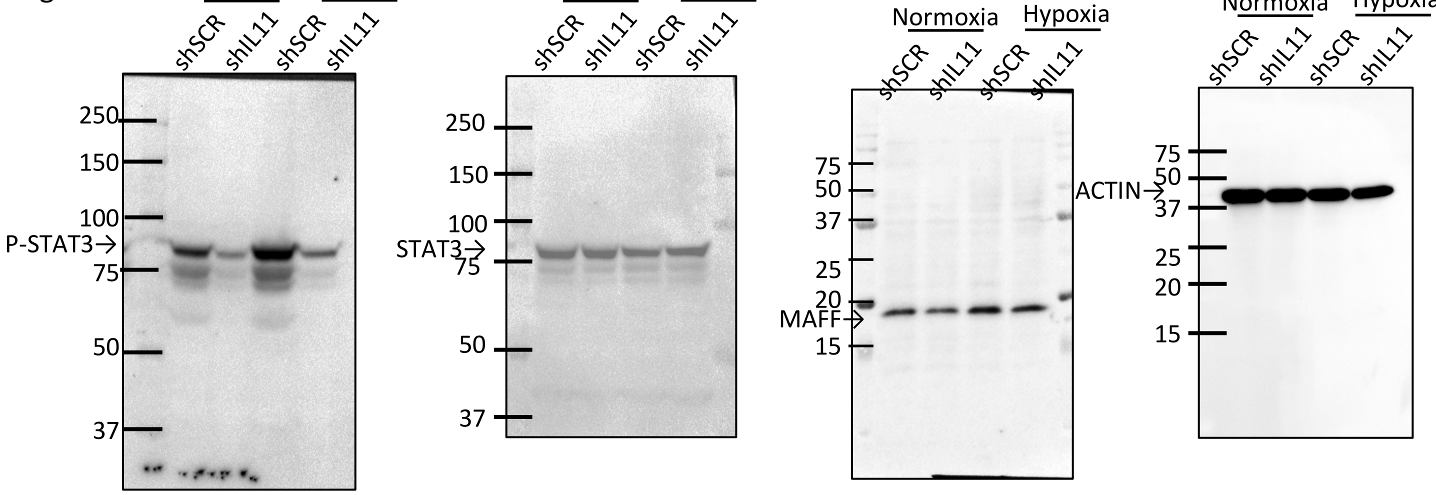

Figure 6e

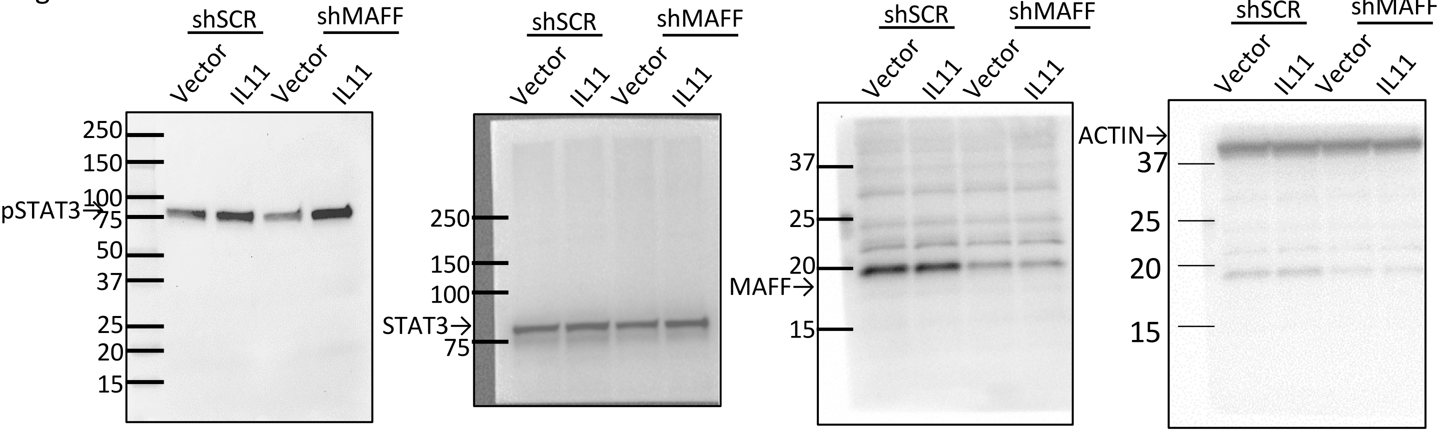

Figure 7b

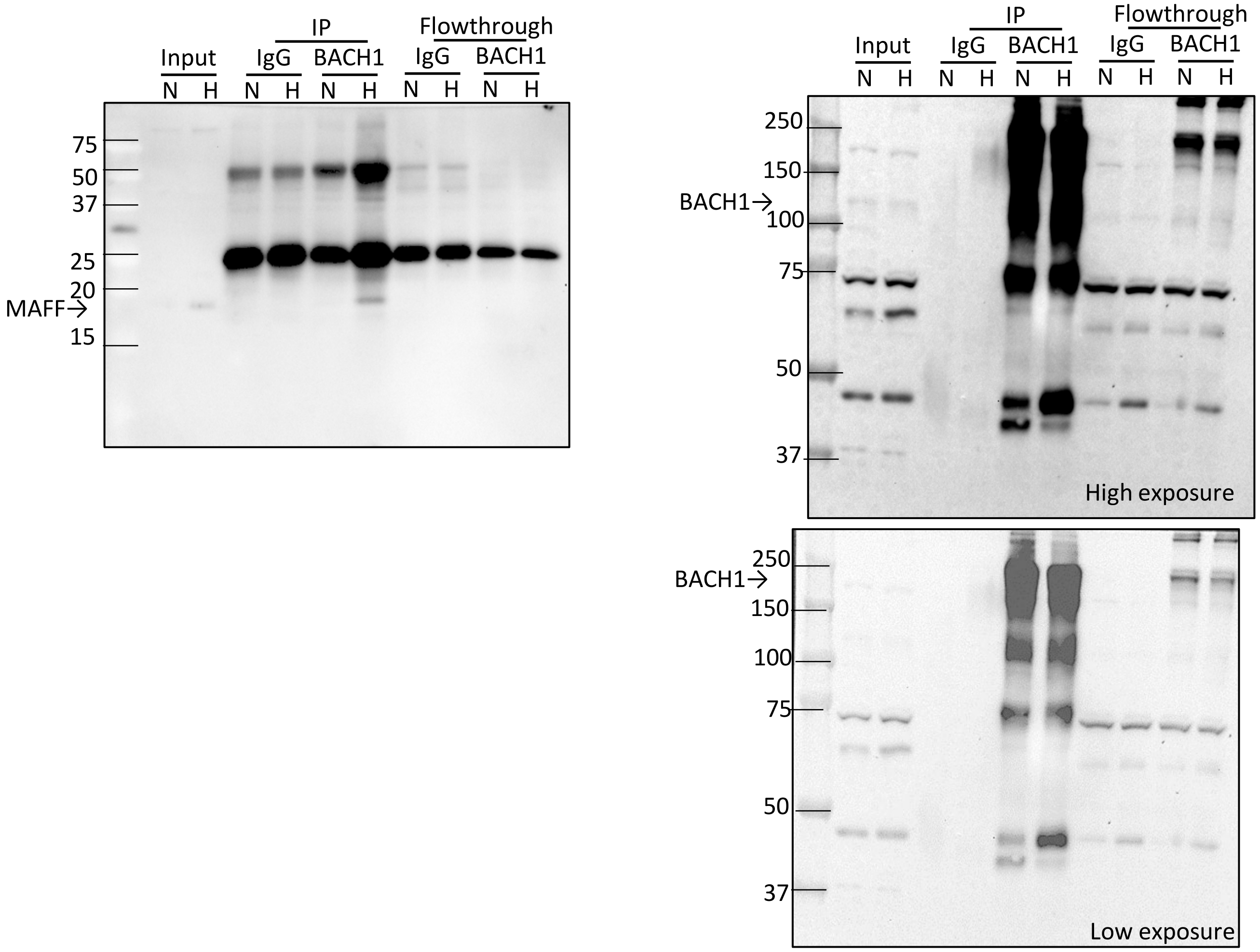

Figure 7d

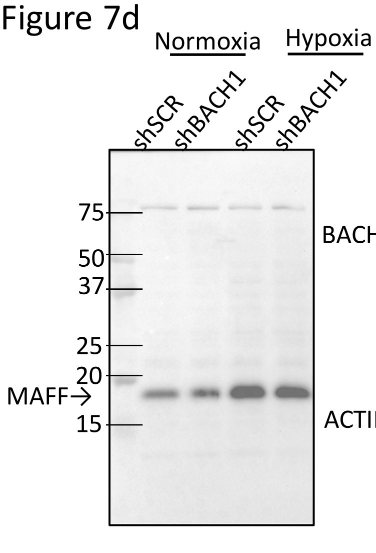

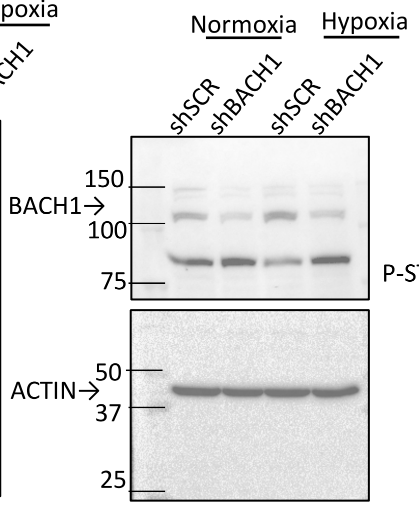

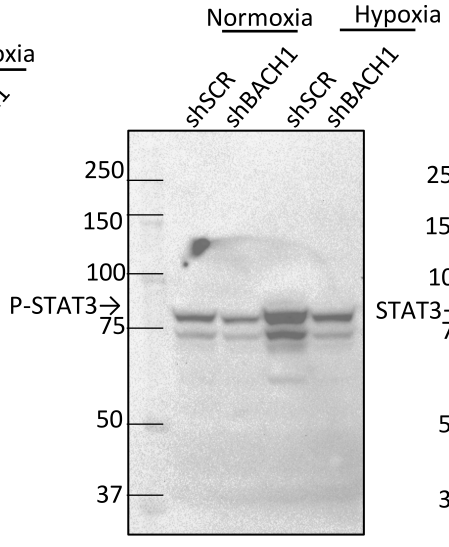

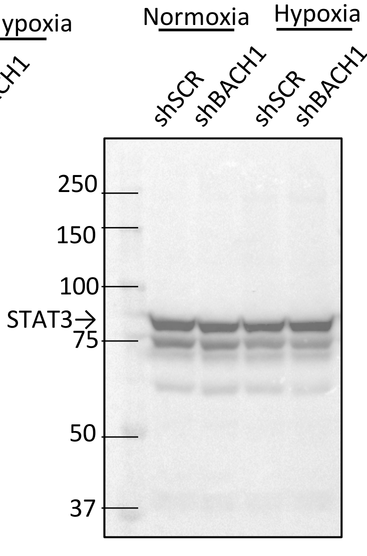

Supplementary Figure 2c

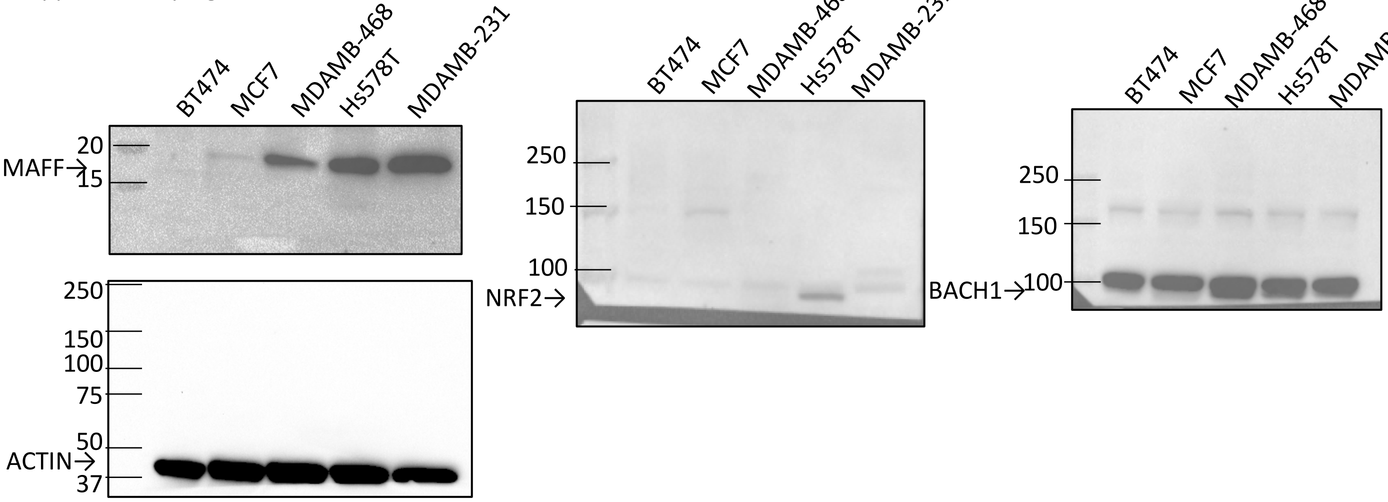

Supplementary Figure 2d

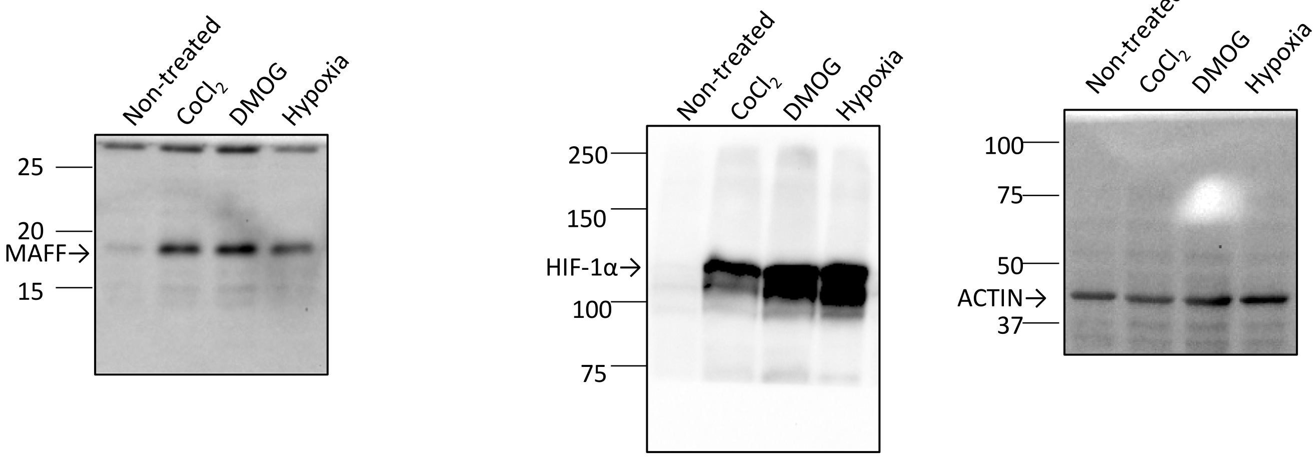

Supplementary Figure 2f

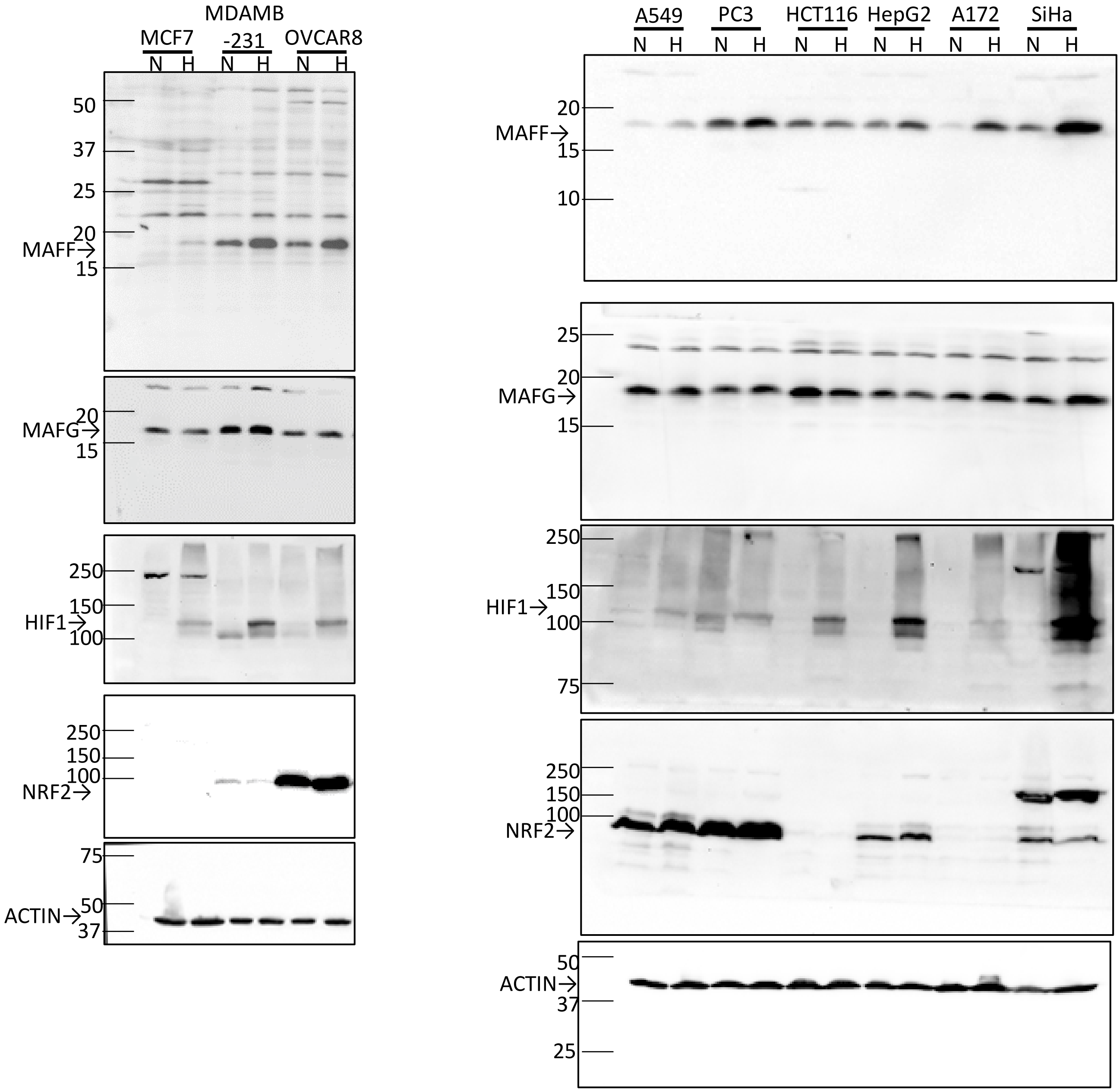

Supplementary Figure 2h

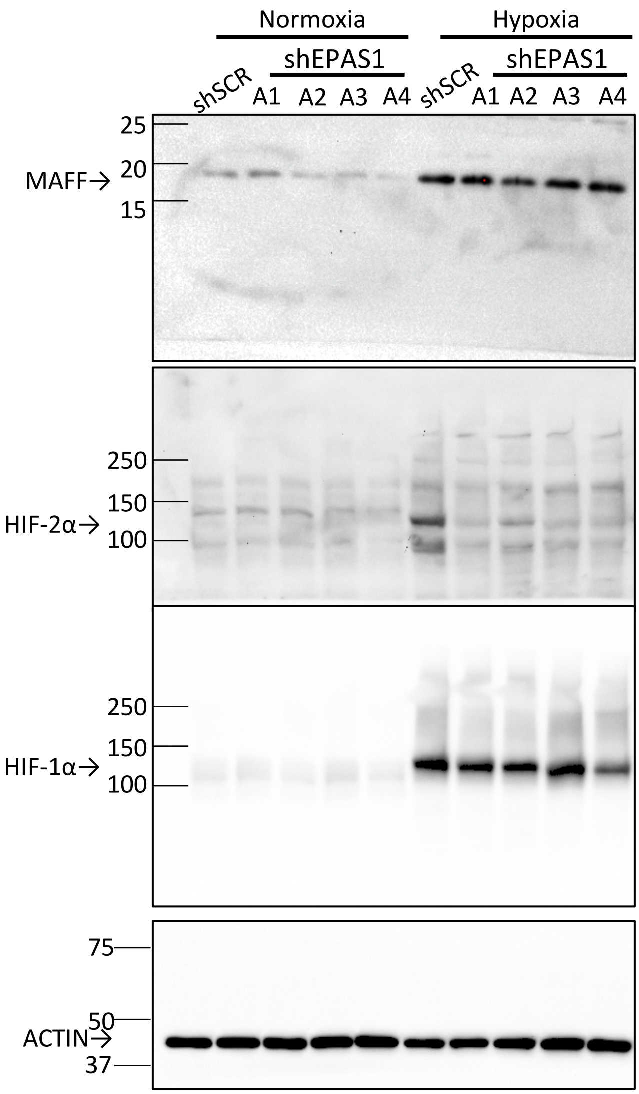

Supplementary Figure 3e

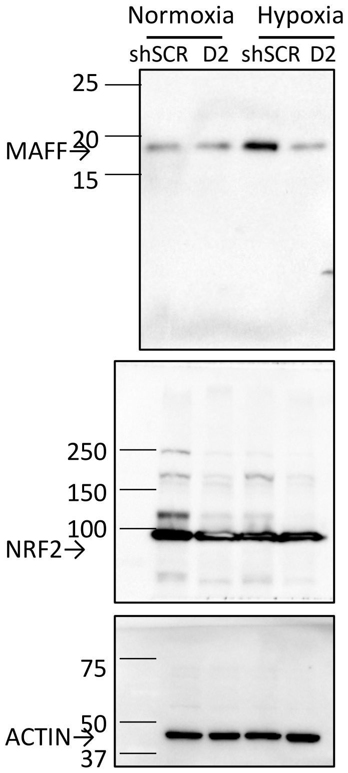

Supplementary Figure 3f

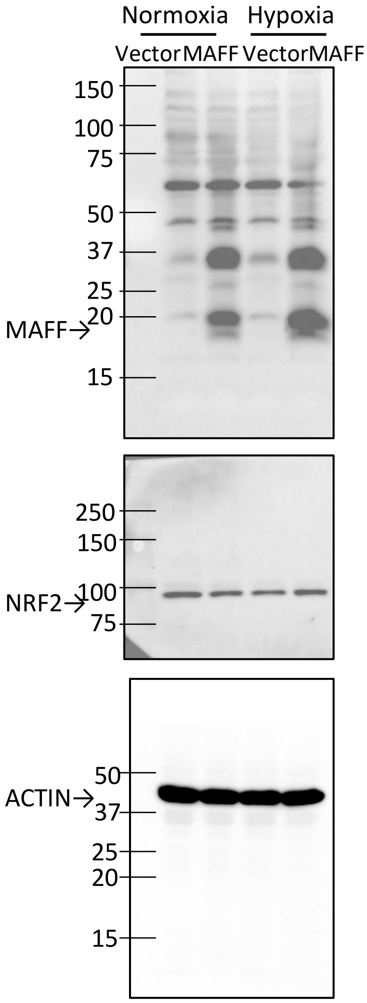

Supplementary Figure 7e

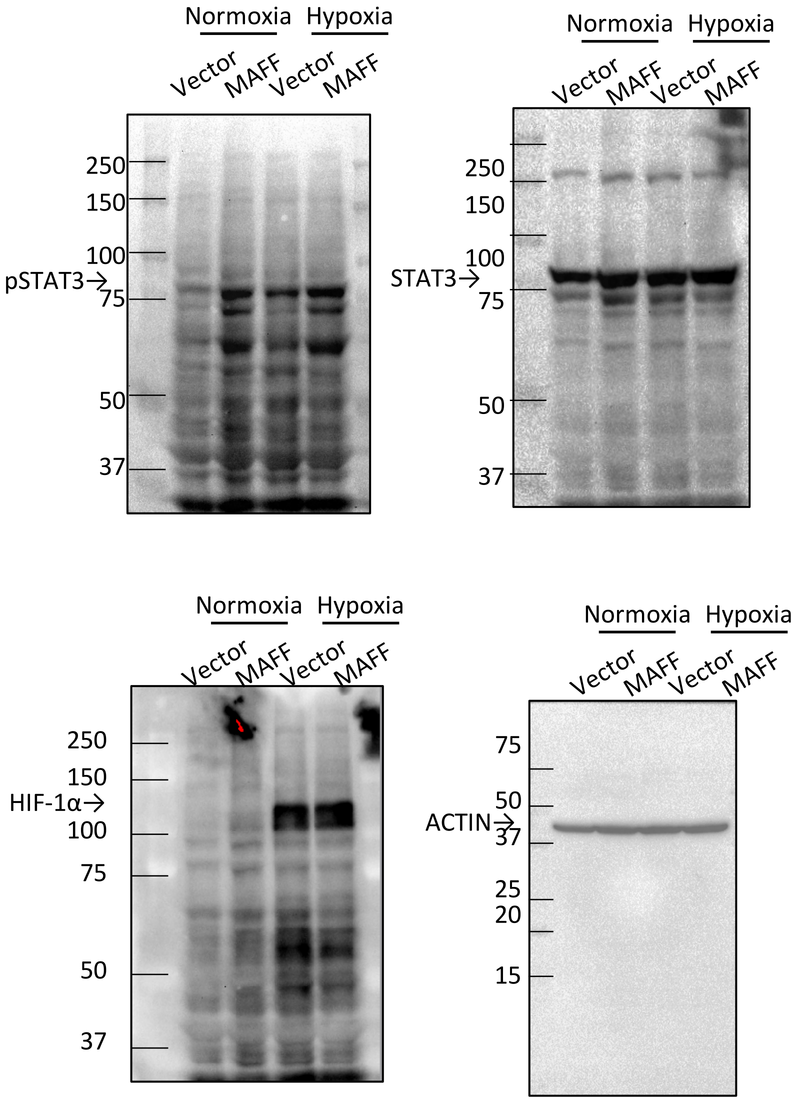

Supplement: Supplementary file 11 — Source data [file 41467_2021_24631_MOESM11_ESM.zip › Data Source/Western raw data.pdf]
